# Sport-Related Injuries in Portuguese Padel Practitioners and Their Characteristics

**DOI:** 10.3390/medicina61091707

**Published:** 2025-09-19

**Authors:** Ricardo Maia Ferreira, Luís Gonçalves Fernandes, Luís Diogo Campos, Rui Soles Gonçalves

**Affiliations:** 1 Social Sciences, Education and Sport Department, Polytechnic Institute of Maia, N2i, 4475-690 Maia, Portugal; rferreira@ipmaia.pt (R.M.F.); lfernandes@ipmaia.pt (L.G.F.); lcampos@ipmaia.pt (L.D.C.); 2 Scientific-Pedagogical Unit of Physiotherapy, Coimbra Health School, Polytechnic University of Coimbra, 3046-854 Coimbra, Portugal; 3 Sport Physical Activity and Health Research & Innovation Center (SPRINT), 4960-320 Melgaço, Portugal; 4 Health & Technology Research Center (H&TRC), Coimbra Health School, Polytechnic University of Coimbra, 3045-043 Coimbra, Portugal

**Keywords:** Padel, Portugal, epidemiology, sport injuries, musculoskeletal

## Abstract

*Background and Objectives:* Padel is becoming an increasingly popular sport, yet epidemiological data on sport-related musculoskeletal injury in Portuguese practitioners are limited. This study aimed to provide a comprehensive epidemiological analysis of Padel practitioners in Portugal to fill this gap. *Materials and Methods*: A nationwide self-reported questionnaire was administered to Portuguese Padel practitioners. The questionnaire covered sociodemographic, occupational, and health-related items. *Results*: Overall, 69.2% of respondents reported at least one lifetime Padel injury (mean 2.1 ± 1.2 injuries per injured athlete). Estimated injury incidence was 3.4 ± 4.7 injuries per 1000 h of exposure for the total sample (6.1 ± 4.9 per 1000 h among injured athletes). Tendinous (35.6%) and muscular (26.1%) injuries predominated. Most affected anatomical sites were the elbow (18.0%), ankle (16.6%), knee (12.5%), and shoulder (10.5%). Injuries most frequently occurred during training (32.9%) and were commonly attributed to fatigue/overload (24.1%). Reported severity was commonly moderate to severe (most frequent absences: 3–4 weeks and 1–3 months); physiotherapy was the principal management approach. Multivariable analyses identified several associations of practical relevance: higher body mass index was linked to greater overall injury risk (and specifically neck injuries); female sex was associated with higher odds of muscular injuries; years of Padel experience and equipment characteristics (e.g., number of overgrips) showed site-specific associations; and instructor supervision correlated with greater warm-up adherence and lower odds of elbow injury. Playing surface also influenced risk patterns. *Conclusions*: Portuguese Padel practitioners in this study experience a substantial burden of predominantly tendinous and muscular injuries concentrated at the elbow and lower limb. Athlete factors, exposure, surface, equipment, and supervision represent modifiable targets for multidisciplinary prevention and rehabilitation strategies.

## 1. Introduction

Padel, also known as Paddle or Paddle Tennis, is a racket sport that originated in Mexico in the 1960s [1,2,3]. It is played on a 20 × 10 m court and typically features an artificial grass or synthetic surface [4]. The court is divided by a central net similar to Tennis (0.88 m in the middle strap, 0.92 m in the posts) and bounded by metal fences on the sides and glass walls on the ends and corners (with the back wall measuring 3 × 10 m, side walls 3 × 2 m, with another 2 × 2 m wall at the end) [4]. The remaining sections of the court consist of two metallic panels (each 3 × 2.59 m) and a gate (2.20 × 0.82 m) [4].

Given these configurations and associated rules, Padel is classified as an intermittent high-intensity sport, which combines high-frequency and low-intensity sporting actions with specific technical, tactical, and physical demands, synthesizing aspects of Tennis and Squash [5]. For example [4]: (1) Padel players can hit the ball after rebounds off the side and back walls; (2) the Padel ball is 6.35 to 6.77 cm in diameter, weighs 56.0 to 59.4 g, and has a lower bounce compared to a Tennis ball (bouncing between 135 and 145 cm after a drop of 2.54 m); (3) Padel players use a short-handled racket (not exceeding 45.5 cm) with a flat, perforated head of foam core encased by a covering of fiberglass or carbon fiber, of varied shapes (e.g., diamond, teardrop, and round); (4) the serve is performed by bouncing the ball and hitting it while it is below hip level.

Due to these characteristics, Padel has become the fastest-growing racket sport, and has experienced a substantial growth in practice across Europe [1]. It is currently practiced in over 130 countries and governed by more than 70 federations [6], including Portugal [7]. The sport’s accessibility, sociability, enjoyment, and perceived health benefits [1,8] have contributed to its widespread adoption, with over 500,000 licensed players worldwide [6], including approximately 27,000 in Portugal (amateur and professional levels combined) [9].

This surge in popularity has been accompanied by a corresponding increase in scientific research interest [10], with more than half of all related studies published in the last four years [11]. The existing literature has explored various topics, such as performance, biomechanics, match analysis, technical and tactical parameters, and players’ physical, physiological, and psychological profiles, as well as epidemiology [1,2,8,10,12,13,14]. The studies have shown that Padel injuries may affect all parts of the body (e.g., upper limbs, lower limbs, and face), and are influenced by personal (e.g., age, sex, training/playing loads, experience, or skill level) and sport-specific (e.g., Padel-related gestures or fitness demands) factors, which may alter their reporting and patterns [1,2,8,10,14,15,16]. These potential risks associated with playing Padel highlight the need for a better understanding of the incidence, prevalence, and nature of injuries among recreational and professional players.

Despite its increasing popularity within the scientific community, research on the epidemiology of Padel-related injuries is still limited [2]. Limited research has explored the epidemiology and nature of Padel injuries, mostly in Spanish [17,18,19,20] or Italian [21,22,23,24,25] contexts. The paucity of data regarding Padel injuries in the literature is a significant limitation, hindering a comprehensive understanding of the sport’s injury landscape. Understanding the epidemiology of injuries in this athletic population is necessary to guide the development of targeted, evidence-based injury prevention strategies, to optimize training programs, and to inform public health initiatives promoting safe participation in the sport. The limited research on Padel injuries restricts the availability of reliable and robust data. To our knowledge, no epidemiological studies on Padel injuries have been conducted in Portugal. Therefore, the objective of this study is to provide a comprehensive epidemiological analysis of Padel practitioners in Portugal to fill this gap.

## 2. Materials and Methods

To achieve the objective, a retrospective, cross-sectional, self-reported study was conducted, following established methodological guidelines [26,27,28,29]. The study followed the Ethical Principles of the Helsinki Declaration [30] and was approved by the Polytechnic Institute of Coimbra Ethics Committee (D28/2024). Before initiating the e-survey (on the front page), the purpose and context of the study, data use and protection rights, eligibility criteria, instructions about how to fill out and complete the e-survey, and the contact for clarifications were explicitly stated. Consent to participate was obtained through an informed consent statement. After agreeing to participate in the study and with the informed consent, the Padel practitioners were granted access to the e-survey.

### 2.1. Participants and Data Collection

In an attempt to ensure the appropriate population sample, the potential participants were reached through the various Padel clubs’ communication channels. At this point, the study’s procedures, objectives, and eligibility criteria were explained. The eligibility criteria for participating in the study were: ability to read and write in Portuguese; being over 18 years old; practicing Padel; signing the informed consent; completing the questionnaire; residing in Portugal (mainland or islands); of any sex or Padel experience. The sample size goal was set at 379 responses, based on 27,000 affiliated Portuguese Padel affiliates [9], with a 95% confidence level, 5% margin of error, and 50% response distribution [31]. To ensure that the sample size goal was achieved, a thank you note and a reminder containing the questionnaire link was sent at two-, four-, and six-week intervals.

### 2.2. Questionnaire

The questionnaire link was shared to the potential participants through the software Google Forms (www.forms.google.com (accessed on 28 August 2025)). Prior to sending the e-survey, the questionnaire was developed by reviewing the most recent literature and pre-tested by the authors evaluating its completion time, design, question order, attractiveness, syntax, clarity, logic, correct question type, and response format. Furthermore, the questionnaire was reviewed by two independent experts with experience in methodology and in the areas of health and exercise, and pre-tested in a representative sample of ten Padel practitioners, where they were able to comment and suggest improvements. The e-survey took 15–20 min to complete and consisted of 48 close-ended questions, which were divided into two main stages (38 sport and sociodemographic-related items, and 10 health disorders and injury-related items—Supplemental File S1):

1. Sport and sociodemographic-related items. This questionnaire phase covered questions regarding age, sex, height, weight, professional physical level, sports background, Padel years and level, Padel competitions/training load and context, and warm-up and cool-down strategies.
2. Injuries and health disorder-related items. In this phase, the practitioners self-reported their health disorders and injuries related to Padel by answering the following question: “Have you experienced any injury related to your Padel practice?” If the answer was a positive response, respondents were asked to specify their frequency, location, type, context, time to return-to-sport, health history, and management. The items in this phase were adapted from a consensus statement on injury registration [32]. As there is no consensus definition of injury by the International Padel Federation [2], injury was defined as any physical damage to a body part, sustained during training or competition, that prevented the participant from training, working, or competing in any way and for any period of time [32,33,34]. Other Padel injury-related data (e.g., types, locations, incidence, and severity of injuries) were established from previous Tennis consensus statements [35,36]. To ensure a proper questionnaire filling, definitions and examples were placed alongside the items, helping to contextualize the readers.

### 2.3. Statistical Analysis

Data from the online questionnaire were entered into a protected database, from which the data were determined and displayed in tabular and graphical formats, using the Microsoft Excel (v. 2508, Microsoft Corp, Redmond, Washington, DC, USA) and the IBM SPSS (v. 26.0 International Business Machines Corporation, Statistical Package for the Social Sciences, Armonk, NY, USA) software. The injury proportion was determined by dividing the total number of participants who experienced at least one injury by the total number of athletes and multiplying by 100. In addition, the injury rate was calculated by dividing the total number of injuries reported in the last 12 months by the total exposure time to risk (defined as 1000 h). The total exposure time was estimated by adding the average monthly training hours by the average monthly number of competitions (multiplied by the average duration of each competition) and then multiplying by 12. This calculation was performed for the entire sample as well as for the injured athletes subgroup. Furthermore, the average number of injuries per athlete was calculated in two ways: one by dividing the total number of injuries with the total number of athletes, and another by dividing the total number of injuries with the number of injured athletes [37]. As the data was not normally distributed, independent two sample non-parametric Mann–Whitney U (ordinal data) and chi-square tests (nominal data) were conducted. Additionally, Spearman’s rho correlations were performed between the variables. The strength of the relationship was evaluated according to the following criteria [38]: 0.3, 0.5, and 0.6 to be small, medium, and large, respectively. Moreover, logistic regression analyses using the enter model were conducted to examine the associations between the Padel practitioners’ sport/sociodemographic characteristics and the injuries. A significance level of 0.05 was used to define whether a model needed to be reported [39]. Odds ratios (ORs) and their 95% confidence intervals (CIs) were determined for each level of the independent variables. CIs that included 1.0 were considered as not statistically significant [40]. The R^2^ values were interpreted as follows [41]: R^2^ < 2%—very weak; 2% ≤ R^2^ < 13%—weak; 13% ≤ R^2^ < 26%—moderate; and R^2^ ≥ 26%—substantial.

## 3. Results

With the e-survey, 6375 Padel practitioners could be reached and, of them, 455 showed interest in participating in the study (Supplemental Figure S1 illustrates the study’s flow diagram). Of the 426 Padel practitioners who completed the questionnaire, the majority were males (291; 68.3%), with a mean age of 41.7 (±9.8) years, and a BMI of 24.5 (±3.2) kg/m^2^, being employed and reporting a related physical activity level of sitting and walking without physical efforts (167; 39.2%). Before engaging in Padel, 398 (93.4%) participants reported having practiced sports or physical activity, with 253 (63.6%) doing so for over 10 years, averaging 293 (±221) minutes per week, most frequently in Soccer or Tennis (both with 66; 16.6%). Currently, the participants have a mean Padel experience of 4.2 (±3.1) years, training on average 2.7 (±1.3) times weekly, totaling 96.1 (±50.5) minutes. Additionally, they reported playing an average of 4.9 (±4.3) matches monthly at a 4-level (male: 3.6; female: 3.6; and mixed: 3.7). Most of them also reported practicing other sports or physical activities as well (239; 56.1%), with an average weekly training duration of 165 (±98.8) minutes, primarily in gym/weightlifting (128; 53.6%). The majority of the participants trained in the Lisboa region (146; 34.5%), on synthetic material playing surfaces (249; 58.5%), and used Padel-specific footwear (387; 90.8%). There was also predominance among the practitioners in playing on the left side of the field (214; 50.2%) and using their right hand (401; 94.1%). Regarding the characteristics of the rackets used, the majority were light (217; 50.9%), had an outer shell composed of carbon fiber (325; 76.3%), featured a soft core (153; 35.9%), were teardrop-shaped (181; 42.5%), and had a single overgrip (260; 61.0%). Typically, they dedicated less than 10 min to their warm-up routines (283; 66.4%), incorporating three different warm-up strategies/interventions, composed mainly of mobility exercises (280; 31.3%) and often combined with other exercises (namely, jumping/plyometrics, static and dynamic stretching, jogging, and sprinting—60; 17.5%). Similarly, although the majority reported not using cool-down strategies (209; 49.1%), those who did spent less than 10 min (189; 44.4%), performing stretching exercises (166; 76.5%). Furthermore, the Padel practitioners reported being monitored by a Padel instructor (289; 67.8%), but not by a health professional (285; 62.2%). Detailed data on Padel practitioners’ sport and sociodemographic characteristics can be accessed in Supplemental Tables S1 and S2.

Of the 426 Padel practitioners who completed the questionnaire, 295 (69.2%) reported having an injury. Most of them reported experiencing two injuries (39.7%) during their years of Padel practice, with one of these occurring in the last 12 months (50.2%), resulting in an estimated incidence of 3.4 (±4.7) injuries per 1000 h of exposure. Elbow (18.0%) and tendon (35.6%) were the most common localization and injury type. Most injuries occurred during training (32.9%), in either the central defensive (2 and 5 zones) or transition (8 and 11 zones) court zones (11.2–11.9% and 13.2%, respectively), with fatigue being identified as the main contributing cause (24.1%). Injuries were usually moderate to severe in severity (more frequently reported sports absenteeism of 3–4 weeks [23.1%] and 1–3 months [28.8%]), had no prior clinical history (72.5%), and were primarily managed with physiotherapy (48.1%). For more detailed information, refer to Supplemental Figure S2 and Supplemental Tables S3–S5a–c. When comparing Padel practitioners with reported injuries to those without, the sole significant statistical difference found was years practicing Padel (*p* < 0.001). The other variables did not exhibit significant statistical differences (refer to Supplemental Table S1 for additional details).

Statistically significant correlations were also found between injuries and other variables. Padel experience years showed a small negative correlation with injuries in the past year (−0.235, *p* ≤ 0.001), indicating that more experienced players reported fewer recent injuries. All other significant injury-related correlations were small and positive. Specifically, BMI showed a correlation with total Padel injuries (0.117, *p* ≤ 0.05), Padel training duration with 1-year injuries (0.156, *p* ≤ 0.01), total years of play with overall injuries (0.140, *p* ≤ 0.05), and overgrip with 1-year injuries (0.119, *p* ≤ 0.05), suggesting that higher BMI, longer training sessions, more years of playing, and more overgrips in the racket handle were each associated with more injuries. Other non-injury-related variables also showed statistically significant correlations. BMI was the most interconnected variable, with nine significant correlations, followed by racket core (five), and years of Padel experience and performing warm-up (both with four). Moderately connected variables (each with three significant correlations) included Padel training duration, other sports participation, weekly frequency of Padel training sessions, dominant side of the playing field, racket weight, and weekly frequency of Padel competitions. Variables with two significant correlations included number of overgrips, Padel competition level, presence of a Padel instructor, and monitoring by a health professional. Variables with low interactions (only one significant correlation) were sex, age, level of professional physical activity, sports background, dominant hand, type of Padel court surface, and performing cool-down. The remaining variables were assessed but did not show statistically significant correlations. For detailed results, see Supplemental Table S6.

Regarding logistic regressions, eleven statistically significant logistic regression models were identified. Each model included a combination of relevant predictors, associated with injury types and anatomical localizations in Padel players.

Three statistically significant models were identified for different types of injury. For tendinous injuries, the model (R^2^ = 0.029; χ^2^ = 6.34; *p* = 0.012; AUC = 0.567) showed that Padel training duration was a significant predictor. Each additional minute of training was associated with a slight but statistically significant reduction in the odds of sustaining a tendinous injury (OR = 0.99, 95% CI [0.99, 1.00]; *p* = 0.041). In the ligament injuries model (R^2^ = 0.111; χ^2^ = 17.2; *p* = 0.028; AUC = 0.711), two predictors emerged. First, occupational physical activity played a role: compared to sedentary individuals, those who reported “standing and walking with moderate physical effort” had 3.5 times higher odds of injury (OR = 3.51, 95% CI [1.04, 11.86]; *p* = 0.044), and those involved in “standing and walking with heavy physical effort” had nearly 13 times higher odds (OR = 12.97, 95% CI [1.62, 104.10]; *p* = 0.016). Second, the court surface type was influential: players who trained on synthetic surfaces had nearly four times greater odds of ligament injury (OR = 3.95, 95% CI [1.48, 10.57]; *p* = 0.006), and those unaware of the surface had 6.6 times higher odds (OR = 6.60, 95% CI [1.37, 31.86]; *p* = 0.019), compared to those playing on artificial grass. For muscular injuries, the model (R^2^ = 0.020; χ^2^ = 4.07; *p* = 0.044; AUC = 0.563) identified sex as a significant predictor, with female participants showing 75% higher odds of injury compared to males (OR = 1.75, 95% CI [1.02, 3.00]; *p* = 0.042).

For injury localization, eight statistically significant models were identified. Starting with neck injuries, the model (R^2^ = 0.122; χ^2^ = 3.88; *p* = 0.049; AUC = 0.697) indicated that each unit increase in BMI was associated with 34% increased odds of cervical injury (OR = 1.34, 95% CI [1.02, 1.76]; *p* = 0.033). The posterior arm injuries model (R^2^ = 0.249; χ^2^ = 5.80; *p* = 0.016; AUC = 0.798) showed that players who trained more frequently were 2.57 times more likely to report injury (OR = 2.57, 95% CI [1.23, 5.40], *p* = 0.012). For elbow injuries, the model (R^2^ = 0.106; χ^2^ = 19.8; *p* < 0.001; AUC = 0.659) identified two significant predictors: years of Padel experience and instructor presence and monitoring. For experience, reduced injury odds by 13% per year was found (OR = 0.87, 95% CI [0.76, 0.99]; *p* = 0.034). For instructor presence and monitoring, compared to those without instructor supervision, participants training without an instructor had 17.31 times higher odds of injury (OR = 17.31, 95% CI [2.16, 138.65]; *p* = 0.007), while those with an instructor also had significantly higher odds (OR = 9.15, 95% CI [1.22, 68.88]; *p* = 0.032). In the wrist injuries model (R^2^ = 0.199; χ^2^ = 18.4; *p* = 0.001; AUC = 0.819), more years of experience significantly reduced injury risk (OR = 0.64, 95% CI [0.44, 0.94]; *p* = 0.022), whereas exclusively playing Padel increased injury odds nearly fivefold (OR = 4.96, 95% CI [1.09, 22.67]; *p* = 0.039). The court surface again played a role: training on synthetic material was highly protective (OR = 0.07, 95% CI [0.01, 0.40]; *p* = 0.003), and playing on artificial grass showed a protective trend (OR = 0.19, 95% CI [0.04, 1.02]; *p* = 0.052), both relative to those unaware of the surface type. The model for hand and finger injuries (R^2^ = 0.094; χ^2^ = 4.40; *p* = 0.036; AUC = 0.721) indicated that longer training duration was a risk factor, with each additional unit of training time slightly increasing injury risk (OR = 1.01, 95% CI [1.00, 1.02]; *p* = 0.011). For lower back injuries (R^2^ = 0.140; χ^2^ = 6.61; *p* = 0.037; AUC = 0.696), side dominance was protective: players with a left-side dominance had 98% lower odds of injury (OR = 0.02, 95% CI [0.00, 0.41]; *p* = 0.011), while those who predominantly used the right-side had 99% lower odds (OR = 0.01, 95% CI [0.00, 0.21]; *p* = 0.004), compared to those uncertain of their dominant side. In knee injuries (R^2^ = 0.081; χ^2^ = 12.20; *p* = 0.007; AUC = 0.609), two significant predictors were identified: prior sport practice, which was positively associated with injury (OR = 1.00, 95% CI [1.00, 1.00]; *p* = 0.008), and playing surface, where both artificial grass (OR = 0.17, 95% CI [0.05, 0.62]; *p* = 0.007) and synthetic material (OR = 0.20, 95% CI [0.06, 0.70]; *p* = 0.011) were found to be protective. Lastly, the posterior lower leg injuries model (R^2^ = 0.043; χ^2^ = 6.19; *p* = 0.013; AUC = 0.547) showed that each additional year of Padel experience increased the odds of injury by 13% (OR = 1.13, 95% CI [1.03, 1.25]; *p* = 0.012). For more detailed information, see Supplemental Table S7.

## 4. Discussion

The present study found important epidemiological, biomechanical, and behavioral data, which help to elucidate the injury landscape among Portuguese Padel practitioners. Of all responders, nearly 70% reported having sustained at least one injury during their Padel careers, while approximately 80% occurred within the past 12 months, with an average lifetime of 2.1 (±1.2) and a past-year of 1.5 (±0.7) injuries, leading to incidence rates of 3.4 (±4.7) and 6.1 (±4.9) per 1000 h of Padel exposure for the total sample and for injured athletes, respectively. These findings are consistent with other similar literature, which reports career injury prevalence in the range of 60–95% and past 8–12 month prevalence of 40–72%, with incidence rates ranging from three to eight injuries per 1000 h [2,14]. In the present study, most of the injuries reported included ligament (11.5%), joint (11.9%), muscle (26.1%), and tendon (35.6%), with the majority localized in the shoulder (10.5%), elbow (18.0%), knee (12.5%), posterior lower leg (10.2%), and ankle (16.6%). These results are also in accordance with the literature, where the most frequently injured body structures are reported to be tendons, joints, and muscles, with the elbow being the most common injury site, and the knee, shoulder, and lower back also being high-risk locations [1,2,14].

There are some differences between Padel on other similar racket sports. In Table Tennis, the overall injury rate is 23.4%, with an incidence of 10.0 injuries per 1000 h, and with lower limbs, shoulders, spine, knees, upper limbs, and trunk being the most affected anatomical regions, with most of the injuries being of musculoskeletal origin [42,43]. Squash shows an average of 5.5 injuries per 12 months, most frequently affecting the lower limbs, followed by the head and spinal regions, and then the upper limbs, with tendon injuries being the most common, followed by muscle and joint injuries [44]. For Badminton, the mean injury incidence was 3.3 per 1000 h, with the lower limbs accounting for over 50% of cases, including strains, sprains, tendinopathy, and stress fractures [45,46,47]. In Tennis, it is expected that 48.1 injuries per 1000 match exposures will occur, with lower extremities (23.0 per 1000) significantly outnumbering those to the trunk (6.12 per 1000) and upper extremities (17.7 per 1000). These injuries are predominantly muscle and tendon, followed by joint, ligament, and bone. Commonly affected areas include: shoulder (superior labrum anterior-to-posterior tears, internal or subacromial impingement, and rotator cuff tendinopathy/tear); elbow (medial and lateral elbow tendinopathy); wrist (extensor carpi ulnaris tendinitis/subluxation, and carpal ligament sprain); hip (groin muscle strain); knee/thigh (thigh muscle strain, knee ligament sprain/rupture, meniscus tear, and extensor tendinopathy/rupture); ankle (ankle sprain and fracture); and trunk (paraspinal muscle strain, rib muscle strain, lumbar disk degeneration and herniation, and abdominal muscle strain) [48,49,50].

Another feature that distinguishes Padel from other racket sports is the increased occurrence of traumatic injuries, particularly maxillofacial cases [21,51,52]. In Padel, this rate can reach 24.7%, compared to 20.8% in Badminton, 19.5% in Table Tennis, and 13.5% in Tennis [52]. Specifically, in the present study, traumatic injuries accounted for 12.4% and facial injuries represented 0.7% of all injuries (all of these were traumatic in nature). The most frequent etiologies of facial skin wounds and dentoalveolar fractures were attributed to racket impacts against the glass wall of the Padel court, either when players attempted to hit the ball near the glass or when they threw the racket against the glass in an act of nervousness [21]. Moreover, ball and racket impacts represented 96% and 4% of all eye-related injuries, respectively [51].

Comparative statistical analysis identified total years of Padel practice as the sole significant predictor of injury reporting (*p* < 0.001). Notably, years of practice exhibited both detrimental and protective influence on injuries occurrence. On the one hand, it is expected that the more years practicing Padel, the more time the player is exposed to the risks of playing the sport, justifying the positive correlation found (r = 0.140; *p* ≤ 0.05). On the other hand, Padel experience years were negatively correlated with injuries in the past year (r = −0.235; *p* < 0.001), suggesting that more experienced players tend to have less recent injuries. This may be explained as experienced athletes have better and more refined sport-specific technical skills, increased chronic sports adaptations, and musculoskeletal resilience, derived by their progressive and cumulative exposure to exercises, training, loads, movements, and stress [16,53]. For example, novice Padel players tend to rely more on actions associated with a higher risk of injury, such as groundstrokes (24%), stroke-like serves (21.5%), and lobs (11.4%), whereas professional players employ volleys (25.1%), wall strokes (22.9%), and smashes (4.8%) more frequently [54]. Furthermore, more experienced players may have the ability to predict or anticipate Padel-specific movements by recognizing visual signals (pre-cues) that occur before the ball is hit [55]. This reflects in fewer errors, superior court positioning, and tactical proficiency [56]. Therefore, expert and novice players may present different techniques, where novice Padel players tend to perform more repetitive shoulder elevations combined with abduction and external rotation movements, along with frequent elbow contractions-elongations (eccentric loads) and explosive supination-pronation movements, and repetitive ulnar-radial deviations and rapid torsional actions at the wrist [15]. Moreover, fatigue measurements revealed average values of 131.7 ± 16.3 bpm and 3.2 ± 2 RPE for first national category players, 156.4 ± 15.6 bpm and 5.9 ± 1.7 RPE for second national category players, and 150.8 ± 14.4 bpm and 5.1 ± 1.7 RPE for third national category players, suggesting that more experienced players possess a greater knowledge of the sport, employing enhanced tactical awareness to resolve specific situations effectively [57]. Therefore, these sporting adaptative behaviors, refined techniques and skills, and specialized and optimized physical conditioning, present in experienced athletes, may explain the protective effect against new injuries in the short-term.

Higher experienced Padel practitioners were associated with lower odds of elbow (OR = 0.87, 95% CI [0.76, 0.99]; *p* = 0.034) and wrist (OR = 0.64, 95% CI [0.44, 0.94]; *p* = 0.022) injuries—the only exception being in posterior lower leg injuries (OR = 1.13, 95% CI [1.03, 1.25]; *p* = 0.012). Conversely, higher training load athletes had an increased higher odds of sustaining posterior arm (OR = 2.57, 95% CI [1.23, 5.40]; *p* = 0.012) and hand/finger (OR = 1.01, 95% CI [1.00, 1.02]; *p* = 0.011) injuries—but not for tendinous injuries (OR = 0.99, 95% CI [0.99, 1.00]; *p* = 0.041). This inverse association between Padel experience and training volume is also demonstrated in the correlation analysis, where years of experience did not correlate with weekly training frequency (r = −0.062; *p* = 0.287) or training duration (r = 0.009; *p* = 0.872)—only between training weekly days and training duration (r = 0.269; *p* ≤ 0.001). For example, Belmar-Arriagada et al. [58] observed that less experienced Padel athletes are more prone to injuries during training, whereas experienced players are more prone to injuries during competition (63.6% training versus 36.4% competition). Moreover, Valério et al. [59] reported that injuries in lower-level Padel athletes occur more frequently at the beginning or middle of training/competition, while more experienced athletes are more likely to sustain injuries toward the end. Additionally, Pérez et al. [16] found that high-level players (ranked 1–50) experienced less severe injuries (1–28 days) primarily of muscular origin, whereas lower-level players (ranked 51–200) more frequently sustained severe injuries (>28 days) predominantly of tendon origin. Due to their lower metabolism, tendon tissues require longer exposure to loads to achieve adaptations compared to muscle tissue [60]. Consequently, novice Padel athletes may exhibit fewer adaptations to sport-specific demands because of their limited experience, poorer physical conditioning, and fewer hours of exposure. Conversely, it can be inferred that higher-ranked players, benefiting from better physical preparation and a greater number of matches played, possess more adapted tendons and, as a result, their injuries are more likely related to tiredness, predominantly affecting muscle tissue [16,22,59]. Reflecting on the data, these findings align with the proposed “training-injury prevention paradox”, which attributes a higher injury risk less to high absolute training volumes and more to inappropriate, unplanned, excessive, and abrupt load progressions, particularly pronounced in unskilled physically unprepared novice athletes [61]. Therefore, it is recommended that novice Padel practitioners engage in Padel sport-specific technical–tactical training/monitoring, complementary physical conditioning, and briefer Padel training/competitions sessions to gradually improve their sport gesture execution, fitness, and load tolerance, helping to reduce the risk of injuries [14,17,19,58,62].

Padel experience may have also influenced responses to other variables. Most of the “do not know” respondents were inexperienced (of the 138 “do not know” responses, 55% where from practitioners with three or less years of Padel experience). Therefore, certain regression models that included playing surface (namely ligament, wrist, and knee injuries) and field side (for lower back injuries) should be interpreted with caution. For example, synthetic surfaces and artificial grass appeared to have a protective effect against some injuries compared to respondents who were uncertain about the surface. Specifically, synthetic surfaces were associated with reduced odds of wrist (OR = 0.07; *p* = 0.003) and knee injuries (OR = 0.20; *p* = 0.011). Likewise, artificial grass was protective against both wrist (OR = 0.19; *p* = 0.052) and knee injuries (OR = 0.17; *p* = 0.007). Notably, 50% of those responding “do not know” to surface type had only 1–3 years of experience, suggesting that these protective effects may reflect Padel player (in)experience rather than the surface characteristics per se.

An exception to the general trend was identified in ligament injuries, where training on synthetic surfaces was associated with a significantly higher risk compared to artificial grass (OR = 3.95, 95% CI [1.48, 10.57]; *p* = 0.006), suggesting a potential surface-specific risk factor. This elevated risk might be explained by the anatomical localization and biomechanical context of these injuries. The majority of ligament injuries were reported at the ankle (*n* = 12; 35.3%) and knee (*n* = 13; 38.2%) and were commonly associated with forward (*n* = 4; 11.8%) or lateral (*n* = 6; 17.7%) movements. This pattern is further supported by movement-specific injury data. Among ankle injuries, forward (*n* = 5; 10.2%) and lateral movements (*n* = 13; 26.5%) were the most frequently reported mechanisms. Similarly, for knee injuries, forward (*n* = 3; 8.1%) and lateral (*n* = 9; 24.3%) movements also predominated. Additionally, 10.2% of respondents reported posterior lower leg injuries, with the majority being musculotendinous-related (39.7%). In fact, studies that explored Padel movement patterns showed an average distance of 8–12 m per point, 2500–3500 m per game, with short-distance sprints (0.60 and 1.70 m/s) in different directions—predominately forward and sideward [8]. Padel is characterized by high-intensity repetitive changes in direction, jump shots, multidirectional movements, and rapid accelerations and decelerations, requiring athletes to exhibit precise coordination to rapidly adjust their stance in response to ball trajectory, respond to the sport movements (e.g., center of mass changes and take-off and landing phases), and generate substantial lower-limb power to efficiently transfer upper-body momentum [1,2,14,15]. This kinetic chain, performed on firmer or more rigid synthetic surfaces (which makes sliding much more difficult) and combined with higher body weight (74.9 ± 13.2 kg in injured athletes versus 72.6 ± 14.0 kg in non-injured athletes), may amplify torsional shear forces and contribute to increased joint loading and ligament stress, thereby elevating the risk of injuries [14,15,49].

These injury risk patterns may be further evidenced when fatigue is associated. As fatigue accumulates, proprioceptive acuity and neuromuscular control are diminished, thereby increasing the likelihood of muscle, joint, and ligament injuries [16,18,59]. In the present study, fatigue represented 24.3% (1^st^) and 14.3% (2^nd^) of the perceived causes of knee and ankle injuries. Overall, fatigue was the most frequently reported perceived cause of injury among Padel practitioners (*n* = 71; 24.1%). As Padel is played in a small court size, at a quick pace, leading to faster and more intense gameplay, it has been shown that fatigue has a significant role, with athletes self-reporting substantial increases in both physical and mental fatigue after a Padel game [14]. Particularly, it is estimated that an overall match playtime of ~45% (with a 0.84 work/rest ratio), 12.5 to 13.5 s point duration (with most points scored between the fifth and eleventh second of the rally), approximately one stroke per second pace (0.70 to 0.80 shots per second, mean 10 hits per point), results in mean VO_2_ values ranging from 23 to 56 mL/kg/min and blood lactate concentrations of 1.8 to 3.4 mmol/L [10,12,13,16,63]. This translates into RPE values between 3.2 (±2.0) to 5.1 (±1.7)—depending on the athlete Padel level [12]. This fatigue-related vulnerability can also explain the increased injury risk found in specific populations. Specifically, Padel practitioners who engage in physically demanding labors (OR = 3.51, *p* = 0.044 for standing and walking with moderate physical effort; and OR = 12.97, *p* = 0.016 for standing and walking with heavy physical effort) and those who practice Padel exclusively (OR = 4.96; *p* = 0.039) had higher odds of sustaining ligament and wrist injuries. Therefore, lifestyle variables should also be considered when analyzing these variables’ associations [64]. These findings suggest that performance enhancement and injury prevention programs should be designed to be tailored to the specific loads, court surfaces, and equipment characteristics unique to each playing environment and personal/athlete profile.

Although lower back injuries accounted for only 3.4% of the cases in the present study, they are consistently reported as one of the most common injury localizations in Padel [2]. However, this data is partially unexpected, considering that Padel athletes exhibit greater lumbar isometric strength compared to the general population, potentially conferring a protective effect [14]. The present study found that the dominant playing side might influence the likelihood of lower back injuries. This association can, in part, be explained by the relationship between hand dominance and court positioning. Usually, the left player takes more hits than the right player, playing more offensively [65]. For two right-hand-dominant couples, the more dominant or powerful player is typically positioned on the left side of the court, as they can execute the stroke with their dominant hand, allowing both players to keep their dominant forehands oriented toward the center of the playing area, thus maximizing offensive coverage with their most effective stroke [65]. Conversely, when a left-handed player is present, they are typically positioned to the right side for the same biomechanical advantage and defend the center line more effectively [65]. Thus, the court right side may be occupied by either a right- or left-handed player, depending on team composition. In our dataset, the majority of left-handed players were positioned on the court right side (left side: *n* = 1, 4.2%; right side: *n* = 23, 95.8%), whereas the majority of right-handed players were positioned on the left side (left side: *n* = 213, 53.5%; right side: *n* = 185, 46.5%). Although both groups exhibit similar technical game patterns throughout a match (*p* = 0.330), their effectiveness rates differ significantly (*p* = 0.012) [66]. Specifically, left-handed players score more points using smashes (63.3% versus 40.7%) but commit more errors when playing off the wall (37.7% versus 19.5%), whereas right-handers make fewer securing errors (11.2% versus 8.2%) and perform more continuity actions (84.9% versus 79.9%) [66]. Overhead strokes in Padel require greater power from trunk muscles, where these strokes are characterized by an early loading phase characterized by hyperextension of the spine coupled with a twist of the trunk, and a late phase with rapid and violent release of the elastic energy to hit the ball as hard as possible [15]. Therefore, as players positioned on the right are often required to compensate for suboptimal overhead stroke mechanics, they generate an increased trunk explosive rotational force [1]. Considering that, out of an average 4700 s of total game time, about 1600 s are real time play, the number of strokes per match can reach approximately 1300 [12]. So, over time, these repeated high-velocity rotational movements may result in dyskinesis and place excessive mechanical stress on the posterior spinal structures, potentially increasing the risk of low back pain [50]. On the other hand, left-side players exhibited higher rates of accelerations and decelerations, as well as greater total distance covered per match compared to right-siders, resulting in increased physical demands due to these technical–tactical actions [67]. This may be balancing the occurrence of injuries, therefore showing similar prevalence. However, a deeper analysis of the data suggests this may be due to a pattern similar to that observed in the playing surface, in which player experience exerts a more substantial influence on injury risk than court positioning alone, as it was found that Padel practitioners who were aware of their dominant playing side reported significantly lower odds of sustaining lower back injuries compared to those who were not (OR = 0.02, *p* = 0.011 for left; OR = 0.01, *p* = 0.004 for right).

Of the personal characteristics analyzed, BMI and sex were the most relevant predictors of injury. Regarding BMI, it was the only variable directly associated with overall injury incidence in Padel (r = 0.117; *p* ≤ 0.05). Specifically, a higher BMI was linked to an increased likelihood of neck injuries (OR = 1.34, 95% CI [1.02, 1.76]; *p* = 0.033). Although a direct causal link between BMI and neck (or other) injuries is difficult to establish, the most likely contributing factors are unhealthy lifestyle and behavioral determinants (e.g., physical inactivity, poor diet, excessive alcohol consumption, smoking, stress, and insufficient sleep) [19,20,22]. However, based on the study data, this association may also be partially explained by Padel-specific factors. In the present study, Padel players who had a higher BMI also tend to have more years of experience (r = 0.181; *p* ≤ 0.01) and longer training durations (r = 0.148; *p* ≤ 0.05). Additionally, they commonly used heavier rackets (r = 0.247; *p* ≤ 0.001), which were often balanced with additional overgrips (r = 0.195, *p* ≤ 0.001 for BMI versus overgrips; and r = 0.158, *p* ≤ 0.01 for racket weight versus overgrips). This combination of greater training exposure, sport-specific equipment (e.g., heavier, stringless rackets), and compensatory adaptations may contribute to increased biomechanical demands on the elbow, shoulder, and cervical–thoracic–lumbar spine (particularly with high-intensity repetitive concentric and eccentric loading during explosive accelerations and decelerations involving ball impact—specifically associated with overhead actions) [2,15]. Due to the Padel sport-specific characteristics, it is estimated that the majority are overhead actions, including 25–35% volleys, 20–30% directs, 15–25% overhead groundstrokes (smashes and bandejas), 15–20% wall shots, and 10–15% lobs [13,66]. It should be noted that Padel players in this study most frequently reported shoulder and elbow injuries with either the bandeja and smash actions (between 9 and 16%), with most injuries occurring in the central defensive (22.1%) or transition (26.4%) court zones, where it is expected that such explosive actions are most commonly executed [13]. Consequently, as expected [62], the data also revealed that those with a higher weekly training volume reported injuries more frequently (98.1 ± 56.2 min versus 91.8 ± 34.1 min, respectively), reinforcing the interpretation that the elevated odds of neck injuries observed in players with higher BMI may reflect multivariable cumulative loading effects rather than excess body weight alone.

Another variable related with BMI, and relevant to injuries, was sex (r = 0.339; *p* ≤ 0.001). Similarly to what has been reported in the literature [1,2,12], this study included a higher proportion of male (*n* = 291; 68.3%) than female participants (*n* = 135; 31.7%). Within the context of muscular injuries, sex was the sole significant predictor, with female participants demonstrating significantly higher odds of sustaining such injuries compared to their male counterparts (OR = 1.75, 95% CI [1.02, 3.00]; *p* = 0.042). This can be attributed to differences in anthropometric characteristics as well as the Padel sport-specific physical demands. Player height and arm span are crucial in Padel. Taller players, with greater arm span and muscle mass, are able to execute more powerful strokes [14,56]. Additionally, the literature indicates that, overall, female Padel players tend to exhibit lower levels in performance-related outcomes than their male counterparts [10], particularly in strength-related measures (e.g., handgrip strength, bench press, leg press, leg extension, leg curl, lat pulldown, overhead press, shoulder press, and countermovement, squat, and Abalakov jumps) [68,69]. In the present study, male players exhibited superior anthropometric characteristics compared to females (height: 1.78 ± 0.06 versus 1.64 ± 0.06 m; weight: 80.1 ± 11.0 versus 61.5 ± 8.56 kg; BMI: 25.2 ± 3.06 versus 22.8 ± 2.74 kg/m^2^); thus, it is expected to observe such a biomechanical advantage. Furthermore, on average, male Padel players record approximately 4600 s of total game time (1500 s of real time), with sets lasting 2300 s and points 11 s, resulting in roughly 1200 hits per match (8.9 hits per point). In comparison, female Padel players average 4800 s of total game time (1800 s of real time), with sets lasting 2600 s and points 13 s, culminating in approximately 1300 hits per match (10.5 hits per point) [12]. Out of these hits, technical–tactical analyses indicate that professional men’s Padel has a higher frequency of fence shots, backhand volleys, drop shots, viboras, and fake smashes, whereas professional women’s Padel involves more backhands, double walls, bandejas, and fake smashes [56]. In fact, these actions are predictable but different in males and females Padel practitioners. In men, the use of smash, volley, bandeja, direct, back wall, back wall lobs, and direct lobs followed a foreseeable pattern up to eight lags, whereas women described predictable interactions for volley, bandeja, direct, lobs, and direct lobs up to five lags and for smash and back wall, up to four lags [70]. Specifically, lobs may represent 50% of total back-court shots in men’s matches while, in women’s Padel, the lob is used in 85.4% of the back-court shots, making it one of the most used to achieve offensive positions [13]. This may contribute to sex-related differences in rally patterns, as 50.7% of women’s rallies involve no net exchange, compared to 65.9% of men’s rallies in which the servers maintain the net position [71]. These technical–tactical differences are probably the cause of the increase in playing time, duration of the point, and hits in the women’s category [10,13]. Therefore, these biomechanical and gameplay differences, combined with other intrinsic factors (such as joint laxity, menstrual cycle variations, hormonal influences, neuromuscular control deficiencies, and strength impairments), may increase loading, stress, and fatigue or failure in musculoskeletal structures, ultimately leading to a higher risk of injury [15,16,20,64]. Nevertheless, once again, Padel experience appears to be a potential explanatory factor. In our data, injured male athletes were most frequently classified at competitive level 4 (*n* = 78; 26.4%), while injured female athletes were most commonly classified at level 3 (*n* = 33; 11.2%).

However, it is important to distinguish between age and playing experience. In this study, the mean age was 41.7 (±9.8) where a substantial proportion of inexperienced Padel players (with six or less years of practice) were over 45 years of age. For example, among players with 1–3 years of experience, the most represented age group was 45–49 years (*n* = 47; 21.9%), while for 4–6 years of experience, the most common age group was 50–54 years (*n* = 25; 17.5%). This trend may be attributed to the recent rise in Padel’s popularity, particularly among older individuals who have been less physically active for extended periods and have only recently started being involved in the sport, motivated by goals such as health benefits, improving physical fitness, enhancing mental well-being, or socialization [13,22,58]. As many of these individuals began playing Padel without adequate preparation or supervision, when combined with their age and lower physical conditioning, their risk of injury may increase [14]. For example, the percentage of players over 45 years of age unsupervised by a Padel instructor was higher than those who were supervised (*n* = 61; 44.5% versus *n* = 121; 41.9%). Similarly, the percentage of those over 45 years of age unmonitored by a health professional was also higher than their monitored counterparts (*n* = 15; 47.4% versus *n* = 67; 41.6%). Age alone may also play a direct role, as injured players were, on average, older than their non-injured counterparts (42.5 ± 9.6 versus 39.9 ± 10.0 years, respectively). These findings reinforce what was discussed that injury risk is multifactorial, with personal characteristics, experience level, physical conditioning, and training management all playing interconnected and compounding roles.

Given these findings, the involvement of a multidisciplinary team (such as Padel instructors, fitness coaches, physiotherapists, nutritionists, psychologists, etc.) appears essential—particularly for novice Padel athletes. It is expected that such a team could not only monitor training sessions and design a personalized competition calendar but also assess, correct, and improve sport-specific movements/actions, tailor Padel equipment to individual needs, enhance athlete-specific mental and physical conditioning, and ensure proper preparation and recovery routines (e.g., structured warm-ups and cool-downs, individualized nutrition strategies, and improved sleep quality) [10,17,19,58,59,72,73,74,75,76]. In the present study, athletes supervised by a Padel instructor had significantly lower odds of elbow injury compared to those who only had an instructor present without individualized supervision (OR = 9.15; *p* = 0.032), or those training in clubs without any instructor presence or supervision (OR = 17.31; *p* = 0.007). Moreover, players who had previously practiced other sports before engaging in Padel were found to have higher odds of knee injuries compared to those without such a sport background (OR = 1.00; *p* = 0.008). Additionally, the number of overgrips was associated with recent injuries (r = 0.119; *p* ≤ 0.05), potentially indicating a mismatch between the number of overgrips used and either the Padel racket specifications or the athlete’s hand size. The shape, mass, material, and other structural characteristics of Padel rackets significantly influence their balance and inertial properties, making careful selection essential (for example, since power shots are favored by rackets with higher balance, attacking players tend to prefer diamond-shaped rackets, whereas control-oriented shots are facilitated by lower balance, leading defensive players to favor round rackets) [77]. This may reflect biomechanical errors (especially Padel-specific), improper equipment, or inadequate planning, issues that a multidisciplinary support team could proactively identify, correct, and manage. Further supporting the value of continued professional supervision, most injuries were reported during training sessions (*n* = 97; 32.9%). Furthermore, the study found that those who were supervised by a Padel instructor were more likely to perform warm-up routines than those unsupervised (81.0% versus 78.8%, respectively). Similarly, athletes monitored by a health professional were more likely to engage in cool-down activities compared to those without such support (55.9% versus 48.1%, respectively). Both training- and competition-related activities play an important role in injury management [17,64]. These findings underscore the importance of structured professional guidance in promoting safe training habits, reducing injury risk, and enhancing overall athletic performance.

### Limitations

This study presents several limitations that may affect the interpretation and generalizability of its findings. First, its retrospective and self-reported design introduces potential recall bias (intensified by participants’ varying levels of health literacy and knowledge) and precludes clinical verification. This likely contributed to the variability in responses, as reflected by the large standard deviations observed. Additionally, the use of non-random convenience sampling may have influenced the nature of questionnaire responses and introduced sampling bias. The questionnaire design and operationalization also present notable drawbacks: (a) the use of closed-ended questions limited the collection of in-depth information (qualitative data); (b) due to constraints in the survey software, participants were only able to explore a single injury, limiting a comprehensive understanding of the full scope of injuries experienced over the years. Future research should employ prospective cohort designs using standardized surveillance instruments and objective physical and biomechanical assessments. While the current study offers preliminary guidance, randomized controlled trials are recommended to rigorously design and evaluate Padel-specific injury prevention protocols.

## 5. Conclusions

Portuguese Padel practitioners reported a high lifetime burden of sport-related musculoskeletal injury: 69.2% of respondents experienced at least one Padel injury (mean 2.1 ± 1.2 injuries per injured athlete), with an estimated incidence of 3.4 ± 4.7 injuries per 1000 h of exposure for the total sample (6.1 ± 4.9 per 1000 h among injured athletes). Tendinous (35.6%) and muscular (26.1%) were the most common injuries types, while elbow (18.0%), ankle (16.6%), knee (12.5%), and shoulder (10.5%) were the most frequently affected anatomical areas. Multivariable analyses indicate a multifactorial etiology: athlete characteristics (sex, BMI, years of Padel experience), exposure, training patterns, playing surface, equipment features (e.g., racket/overgrip characteristics), and instructor supervision each contribute to injury risk. Fatigue was commonly perceived as a precipitant.

Taken together, these findings have implications for clinical practice, athlete education, and injury prevention. Priority targets include the prevention of Padel-specific injuries through progressive loading, sport-specific strength and neuromuscular training, structured load-management and season planning, and targeted technique coaching for overhead and transition actions. Equipment and surface considerations should inform individualized racket and footwear selection and training prescriptions. The observed protective association with supervised instruction supports investment in education and routine protocols and reinforces the need for a multidisciplinary support team to monitor and balance performance objectives with health and injury prevention.

## Data Availability

The data presented in this study are available on request from the corresponding author.

## Supplementary Materials

The following supporting information can be downloaded at: https://www.mdpi.com/article/10.3390/medicina61091707/s1. File S1: Study’s Questionnaire; Figure S1: Questionnaire views, participation and completion; Figure S2: Padel court division by injuries occurrences and percentages; Table S1: Padel practitioner’s sport and socio-demographic characteristics; Table S2: Exercises/drills included in the warm-up sessions; Table S3: Padel practitioner’s reported injuries characterization; Table S4: Injury type and localization distribution; Table S5a: Padel practitioner’s variables by injury type and localization; Table S5b: Padel practitioner’s variables by injury type and localization; Table S5c: Padel practitioner’s variables by injury type and localization; Table S6: Spearman correlations between injury, sport, and sociodemographic variables; Table S7: Logistic regressions between the sociodemographic, sport, and injury variables.

## Abbreviations

The following abbreviations are used in this manuscript:

AUC	Area Under the Curve
BMI	Body Mass Index
bpm	Beats Per Minute
CI	Confidence Interval
g	Gram
h	Hour
kg	Kilogram
m	Meter
min	Minute
OR	Odds Ratio
RPE	Rating of Perceived Exertion
s	Second
