# Supplementary material for: Sport-Related Injuries in Portuguese Padel Practitioners and Their Characteristics
Source: Medicina (Kaunas). 2025 Sep 19;61(9):1707. doi: 10.3390/medicina61091707 (PMC12471779; doi:10.3390/medicina61091707)
Supplement: Supplementary file 1 [file medicina-61-01707-s001.zip › Supplemental Figures.pdf]

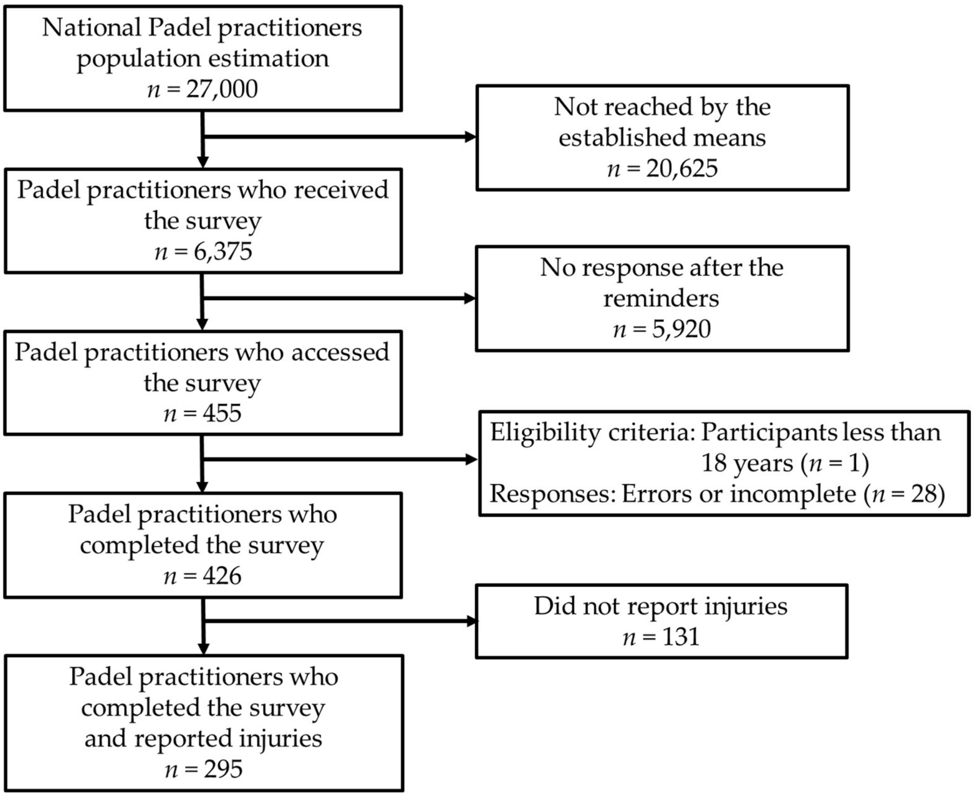

Figure 1. Questionnaire views, participation and completion.

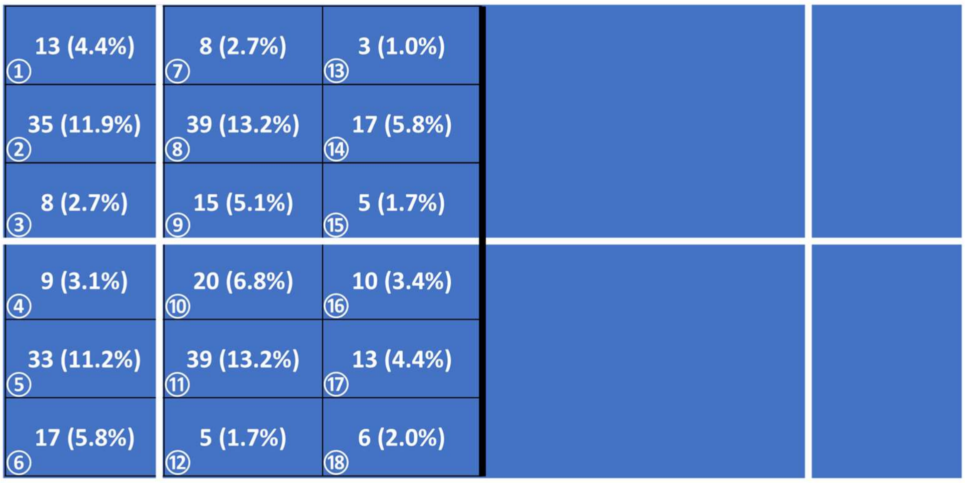

Figure 2. Padel court division by injuries occurrences and percentages (n=295).
