# Supplementary material for: Sport-Related Injuries in Portuguese Padel Practitioners and Their Characteristics
Source: Medicina (Kaunas). 2025 Sep 19;61(9):1707. doi: 10.3390/medicina61091707 (PMC12471779; doi:10.3390/medicina61091707)
Supplement: Supplementary file 1 [file medicina-61-01707-s001.zip › Supplemental File 1 - Questionnaire.pdf]

Principais lesões dos praticantes de Padel

Antes de mais, agradecemos o seu interesse em participar neste estudo.

Este questionário está a ser conduzido no Instituto Politécnico de Coimbra e Instituto Politécnico da Maia, pelo Grupo de Investigação em Saúde e Performance no Desporto e Exercício. Este estudo tem como objetivo fazer o estudo epidemiológico dos praticantes de Padel. Para tal, procuramos participantes que: saibam ler e escrever Português; tenham mais de 18 anos; sejam praticantes de Padel; tenham (ou não) uma lesão desportiva; que não tenham diagnosticada nenhuma deficiência motora, visual ou intelectual.

Todos os métodos utilizados são seguros, não existindo qualquer risco ou experiência dolorosa da sua utilização.

A confidencialidade e anonimato dos dados serão garantidos. A identificação far-se-á por um código, não existindo em nenhum material de referência a dados de identificação.

Após análise de toda a informação recolhida, os dados serão guardados numa base de dados protegida por palavra-passe. Os dados recolhidos são para uso exclusivo do presente estudo, não existindo quaisquer interesses financeiros a motivar o estudo.

A sua participação é voluntária, não existindo nenhuma contrapartida financeira ou de outra natureza, à sua participação. Em qualquer momento, poderá livremente recusar ou interromper a participação no estudo, sem qualquer tipo de penalização por este facto.

Em nome da equipa de investigação do projeto, manifesto os nossos agradecimentos pela sua participação, e manifesto a nossa disponibilidade para quaisquer esclarecimentos adicionais.

* Indica uma pergunta obrigatória

1. Declaro que fui informado do objetivo do questionário e que me foi garantido que todos os dados relativos à identificação dos participantes do estudo são confidenciais e que será mantido o anonimato. Sei que posso recusar-me a participar ou interromper a qualquer momento a participação no estudo, sem nenhum tipo de penalização por este facto. Desta forma, autorizo também a divulgação dos resultados obtidos no meio científico, garantindo a confidencialidade e o anonimato em todos os momentos. *

Marcar apenas uma oval.

- Aceito participar de livre vontade no estudo acima mencionado
- Não aceito participar no estudo acima mencionado
  Avançar para a secção 4 (Agradecimento)

Informação socio-demográfica

Por favor responda às seguinte questões

2. Qual o seu sexo? *

Marcar apenas uma oval.

- Feminino
- Masculino

3. Qual a sua idade (anos)? *

____

4. Qual o seu peso (quilogramas)? *

____

5. Qual a sua altura (metros)? *

____

6. Qual o nível de atividade física no seu local de trabalho? *

Caso seja estudante, considere essa a sua profissão

Marcar apenas uma oval.

- Sentado
- Sentado e a andar, sem grandes esforços
- Sentado e andar, com esforços físicos moderados
- Sentado e a andar, com esforços físicos intensos
- Em pé e a andar, sem grandes esforços
- Em pé e a andar, com esforços físicos moderados
- Em pé e a andar, com esforços físicos intensos

7. Praticou alguma modalidade desportiva ou atividade física antes de iniciar o Padel? *

Por exemplo, correr, ginásio, bicicleta, etc.

Marcar apenas uma oval.

- Sim
- Não

8. Quanto tempo praticou essa modalidade desportiva ou atividade física antes de iniciar o Padel?

Se respondeu <u>Não</u> à questão anterior passe para pergunta seguinte

Marcar apenas uma oval.

- \- 6 meses
- 6 meses - 1 ano
- 2 - 5 anos
- 6 - 10 anos
- \+ 10 anos

9. Em média, por semana, quanto tempo (em minutos) dedicava à prática dessa modalidade desportiva ou atividade física antes de iniciar o Padel?

Se <u>não praticou</u> uma modalidade desportiva ou atividade física passe para pergunta seguinte

____

10. Qual a modalidade desportiva ou atividade física que praticou antes de iniciar o Padel?

Se não praticou uma modalidade desportiva ou atividade física passe para pergunta seguinte

____

11. Pratica alguma modalidade desportiva ou atividade física para além do Padel? *

Por exemplo, correr, ginásio, bicicleta, etc.

Marcar apenas uma oval.

- Sim
- Não

12. Há quanto tempo pratica essa modalidade desportiva ou atividade física para além do Padel?

Se respondeu Não à questão anterior passe para pergunta seguinte

Marcar apenas uma oval.

- \- 6 meses
- 6 meses - 1 ano
- 2 - 5 anos
- 6 - 10 anos
- \+ 10 anos

13. Em média, por semana, quanto tempo (em minutos) dedica à prática dessa modalidade desportiva ou atividade física para além do Padel?

Se respondeu Não à questão anterior passe para pergunta seguinte

____

14. Qual a modalidade desportiva ou atividade física que pratica para além do Padel?

Se não pratica uma modalidade desportiva ou atividade física passe para pergunta seguinte

____

15. Quantas vezes treina Padel por semana? *

____

16. Quanto tempo (minutos) dura em média os seus treinos de Padel? *

____

17. Qual a zona geográfica do seu local de treino habitual? *

Marcar apenas uma oval.

- [ ] Aveiro
- [ ] Beja
- [ ] Braga
- [ ] Bragança
- [ ] Castelo Branco
- [ ] Coimbra
- [ ] Évora
- [ ] Faro
- [ ] Guarda
- [ ] Leiria
- [ ] Lisboa
- [ ] Portalegre
- [ ] Porto
- [ ] Santarém
- [ ] Setúbal
- [ ] Viana do Castelo
- [ ] Vila Real
- [ ] Viseu
- [ ] Madeira
- [ ] Açores

18. Há quantos anos pratica Padel? *

Se praticar à menos de 1 ano coloque o valor 0

____

19. Qual a sua mão dominante na prática de Padel? *

Marcar apenas uma oval.

- Esquerda
- Direita
- Não sabe

20. Usualmente, qual o lado que ocupa no campo Padel? *

Marcar apenas uma oval.

- Esquerdo
- Direito
- Não sabe

21. Qual o calçado que mais usa na sua prática de Padel? *

Marcar apenas uma oval.

- De Padel
- De Ténis
- Não sabe
- Outra: ____________________

22. Qual a forma da sua raquete mais utilizada na prática de Padel? *

Marcar apenas uma oval.

- Redonda
- Lágrima
- Diamante
- Não sabe
- Outra: ____________________

23. Qual o peso da sua raquete mais utilizada na prática de Padel? *

Marcar apenas uma oval.

- Leve (- 370 g)
- Moderada (370 - 385 g)
- Pesada (+ 385 g)
- Não sabe
- Outra: ____________________

24. Qual a composição da sua raquete mais utilizada na prática de Padel? *

Marcar apenas uma oval.

- Fibra de vidro
- Fibra de carbono
- Não sabe
- Outra: ____________________

25. Qual o tipo de preenchimento (núcleo) da sua raquete mais utilizada na prática de Padel? *

Marcar apenas uma oval.

- Rígido
- Mole
- Não sabe
- Outra: ____________________

26. Qual o número de overgrips que utiliza na raquete que mais pratica de Padel? *

Marcar apenas uma oval.

- 0
- 1
- 2
- 3
- 4
- 5
- +5
- Não sabe

27. Qual o terreno de jogo em que mais pratica Padel? *

Marcar apenas uma oval.

- Relva Artificial
- Piso Sintético
- Não sabe
- Outra: ____________________

28. Qual o seu nível de Padel?

Pode selecionar um nível individual e/ou misto

Marcar tudo o que for aplicável.

	1	2	3	4	5	6
**M**	☐	☐	☐	☐	☐	☐
**F**	☐	☐	☐	☐	☐	☐
**Mx**	☐	☐	☐	☐	☐	☐

29. Em média, quantas partidas (momentos competitivos) de Padel realiza por mês? *

Entenda partidas por jogos em ambientes de non-stops, torneios federados, torneios sociais, ...

____

30. Costuma fazer uma ativação antes dos treinos/competições? *

Marcar apenas uma oval.

- ( ) Sim, -10 minutos
- ( ) Sim, 10-20 minutos
- ( ) Sim, 21-30 minutos
- ( ) Sim, +30 minutos
- ( ) Não faço qualquer ativação

31. O que incluí a sua ativação antes dos treinos/competições? *

Pode selecionar mais do que uma opção

Marcar tudo o que for aplicável.

- [ ] Corrida contínua
- [ ] Sprint
- [ ] Exercícios de corrida específicos (por exemplo, skipping, corrida lateral, corrida de costas, ...)
- [ ] Exercícios de mobilidade (por exemplo, rotação dos ombros, rotação dos joelhos, rotação interna e externa da anca, inversão e eversão do tornozelo, ...)
- [ ] Alongamentos dinâmicos
- [ ] Alongamentos estáticos
- [ ] Exercícios de fortalecimento
- [ ] Ativação do core
- [ ] Exercícios de equilíbrio/proprioceptividade
- [ ] Exercícios específicos da modalidade
- [ ] Saltos/pliometria
- [ ] Massagem/Auto-massagem
- [ ] Não faço qualquer ativação
- [ ] Outra: ____________________

32. Qual a principal razão pela qual realiza ativação antes dos treinos/competições? *

Marcar apenas uma oval.

- Rotina
- Concentração
- Fitness/Performance
- Diminuição do risco de lesão
- Redução do nervosismo
- Não faço qualquer ativação
- Outra: ______________________

33. Costuma fazer uma recuperação após os treinos/competições? *

Marcar apenas uma oval.

- Sim, -10 minutos
- Sim, 10-20 minutos
- Sim, 21-30 minutos
- Sim, +30 minutos
- Não faço qualquer recuperação

34. Qual das seguintes estratégias de recuperação após treinos/competições mais utiliza? *

Marcar apenas uma oval.

- Crioterapia
- Massagem
- Recuperação Ativa
- Rolo Miofascial
- Alongamentos
- Pistola de Massagem
- Estimulação Elétrica
- Compressão
- Hidroterapia
- Sauna
- Câmara Hiperbárica
- Repouso Passivo
- Suplementação
- Não faço qualquer recuperação
- Outra: ____________________

35. Qual a principal razão pela qual realiza recuperação após os treinos/competições? *

Marcar apenas uma oval.

- Rotina
- Diminuição do risco de lesão
- Sensação de bem-estar e conforto
- Relaxamento
- Redução da fadiga
- Não faço qualquer recuperação
- Outra: ____________________

36. No seu local de treino existe e usufrui de acompanhamento por parte de um treinador? *

Marcar apenas uma oval.

- Sim e sou acompanhado
- Sim e não sou acompanhado
- Não

37. É acompanhado regularmente por algum profissional de saúde? *

Marcar apenas uma oval.

- Sim
- Não

38. Já teve alguma lesão relacionada à prática de Padel? *

Marcar apenas uma oval.

- Sim
- Não *Avançar para a secção 4 (Agradecimento)*

Lesões relacionadas com a prática do Padel

Por favor responda às seguintes questões segundo as características da sua lesão, ou caso tenha ocorrido mais do que uma, a mais severa

39. Quantas lesões já teve associadas à prática do Padel? *

Entenda lesão como um qualquer dano não intencional ou intencional ao corpo, resultante da participação durante o treino ou a competição, que o/a tenha impedido de treinar, trabalhar ou competir, de qualquer forma e por qualquer período de tempo.

____

40. Nos últimos 12 meses, quantas lesões teve associadas à prática do Padel? *

Entenda lesão como um qualquer dano não intencional ou intencional ao corpo, resultante da participação durante o treino ou a competição, que o/a tenha impedido de treinar, trabalhar ou competir, de qualquer forma e por qualquer período de tempo.

____

41. Indique a tipologia de lesão de sofreu *

Por favor responda à questão segundo as características da sua lesão, ou caso tenha ocorrido mais do que uma, a mais severa

Marcar apenas uma oval.

- Lesão Óssea (fratura, fissura, ...)
- Lesão Muscular (contratura, rutura, ...)
- Lesão Tendinosa (tendinite, tendinose, ...)
- Lesão Fascial (rutura, fasceíte, ...)
- Lesão Articular (entorse, luxação, ...)
- Lesão Ligamentar (ligamentite, rutura, ...)
- Lesão Meniscal (fratura, fissura, ...)
- Lesão Discal (hérnia, protusão, ...)
- Lesão Cartilagínea
- Lesão Nervosa
- Lesão Ocular
- Lesão Auditiva
- Lesão Dentária
- Lesão Nasal
- Bursite
- Contusão/Trauma Cerebral
- Trauma Órgãos
- Corte/Golpe/Laceração/Abrasão/Sangramento
- Algia (dor)
- Outra: ______________________

42. Indique a zona do corpo onde sofreu a lesão *

<u>Por favor responda à questão segundo as características da sua lesão, ou caso tenha ocorrido mais do que uma, a mais severa</u>

Marcar apenas uma oval.

- Face
- Cabeça
- Pescoço
- Coluna Cervical
- Ombro
- Braço (anterior)
- Braço (posterior)
- Cotovelo
- Antebraço (anterior)
- Antebraço (posterior)
- Pulso
- Mão/Dedos
- Tronco
- Coluna Torácica
- Lombar
- Coluna Lombar
- Peito/Costelas
- Abdominal
- Cintura Pélvica (anterior)
- Cintura Pélvica (posterior)
- Inguinal
- Anca
- Coxa (anterior)
- Coxa (posterior)
- Joelho
- Perna (anterior)
- Perna (posterior)
- Tornozelo
- Pé/Dedos

43. A sua lesão ocorreu em que situação? *

Por favor responda à questão segundo as características da sua lesão, ou caso tenha ocorrido mais do que uma, a mais severa

Marcar apenas uma oval.

- Aquecimento (treino)
- Aquecimento (competição)
- Durante o treino
- Durante a competição (1º set)
- Durante a competição (2º set)
- Durante a competição (3º set)
- Durante a competição (+3º set)
- Arrefecimento (treino)
- Arrefecimento (competição)
- Outra: ______________________

44. A sua lesão ocorreu em que zona do campo? *

Por favor responda à questão segundo as características da sua lesão, ou caso tenha ocorrido mais do que uma, a mais severa

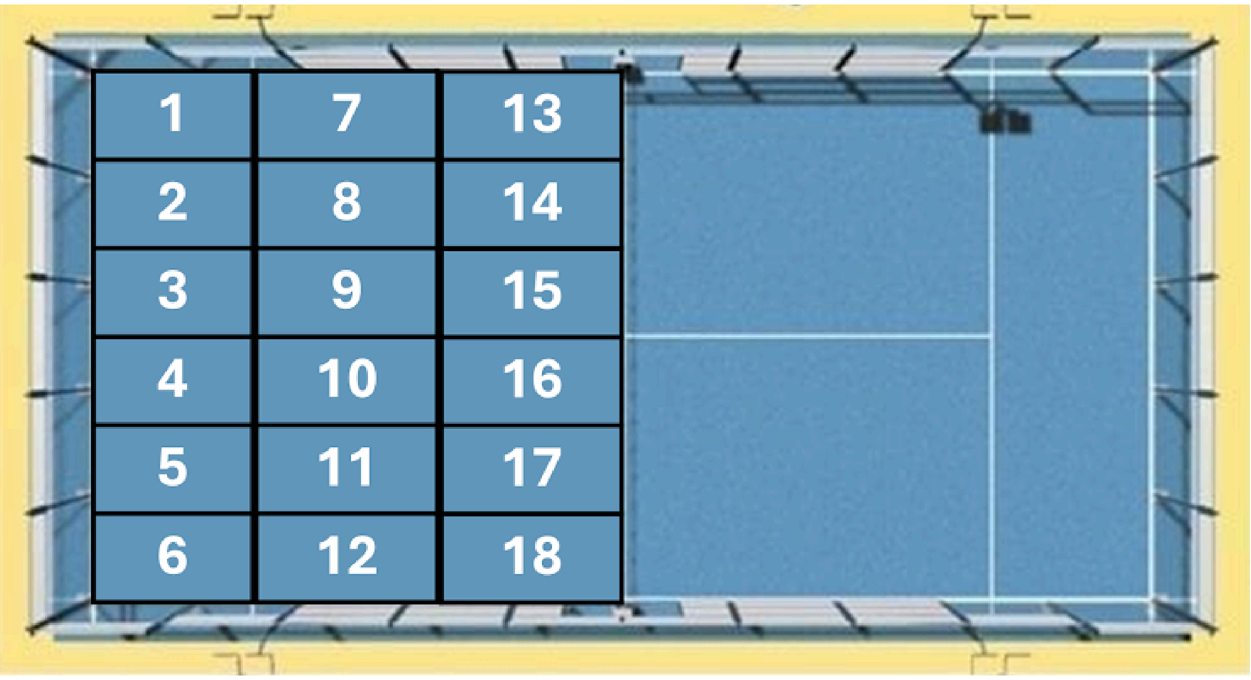

Marcar apenas uma oval.

- 1
- 2
- 3
- 4
- 5
- 6
- 7
- 8
- 9
- 10
- 11
- 12
- 13
- 14
- 15
- 16
- 17
- 18

45. Qual a causa que percepciona ter sido a resultante da sua lesão? *

Por favor responda à questão segundo as características da sua lesão, ou caso tenha ocorrido mais do que uma, a mais severa

Marcar apenas uma oval.

- Fadiga
- Contacto com bola
- Contacto com outro jogador
- Contacto com o vidro de fundo
- Contacto com o vidro lateral
- Contacto com a raquete
- Contacto com a rede
- Contacto com o poste
- Calçado inadequado
- Raquete inadequada
- Infraestruturas/terreno de jogo inadequadas
- Deslocamento frontal
- Deslocamento lateral
- Deslocamento à retaguarda
- Gesto técnico errado da ação de serviço
- Gesto técnico errado da ação de volei
- Gesto técnico errado da ação de bandeja
- Gesto técnico errado da ação de víbora
- Gesto técnico errado da ação de smach
- Gesto técnico errado da ação de lob
- Gesto técnico errado da ação de bola curta
- Gesto técnico errado da ação de chiquita
- Gesto técnico errado da ação de remate
- Desconhecida
- Outra: ______________________

46. De acordo com a sua lesão, quanto tempo demorou o retorno à prática desportiva normal de Padel? *

Por favor responda à questão segundo as características da sua lesão, ou caso tenha ocorrido mais do que uma, a mais severa

Marcar apenas uma oval.

- -1 semana
- 1-2 semanas
- 3-4 semanas
- 1-3 meses
- 4-6 meses
- 7-12 meses
- +1 ano

47. Relativamente à sua lesão *

Por favor responda à questão segundo as características da sua lesão, ou caso tenha ocorrido mais do que uma, a mais severa

Marcar apenas uma oval.

- Foi a primeira que vez aconteceu
- É uma recidiva

48. Como geriu a sua situação? *

Por favor responda à questão segundo as características da sua lesão, ou caso tenha ocorrido mais do que uma, a mais severa

Marcar apenas uma oval.

- Médico (medicação)
- Médico (cirurgia)
- Médico (injeção)
- Fisioterapeuta
- Auto-Medicação/Suplementação
- Auto-gestão (por exemplo, ligaduras, libertação miofascial, fortalecimento, alongamento, ...)
- Terapêuticas não-convencionais (por exemplo, Acupuntura, Osteopatia, Quiropraxia, Fitoterapia, Naturopatia, Homeopatia, Medicina Tradicional Chinesa)
- Repouso
- Sem intervenção
- Outra: ____________________

Agradecimento

Muito obrigado pela sua participação. Sem a sua ajuda este estudo não se podia concretizar.

Este conteúdo não foi criado nem aprovado pela Google.

Google Formulários
