# Supplementary material for: Sport-Related Injuries in Portuguese Padel Practitioners and Their Characteristics
Source: Medicina (Kaunas). 2025 Sep 19;61(9):1707. doi: 10.3390/medicina61091707 (PMC12471779; doi:10.3390/medicina61091707)
Supplement: Supplementary file 1 [file medicina-61-01707-s001.zip › Supplemental Tables.pdf]

Table 1. Padel practitioner's sport and socio-demographic characteristics.

**Variable**	**Total (n=426 (100%)) n(%)**	**Unreported injury (n=131 (30.8%)) n(%)**	**Reported injury (n=295 (69.2%)) n(%)**	***p* Injury vs No Injury**
Age group (years)				0.843*
< 20	5 (1.2)	2 (1.6)	3 (1.0)	
20-24	15 (3.5)	2 (1.6)	13 (4.4)	
25-29	35 (8.2)	10 (8.2)	25 (8.5)	
30-34	60 (14.1)	19 (15.6)	41 (13.9)	
35-39	49 (11.5)	17 (13.9)	32 (10.8)	
40-44	80 (18.8)	28 (23.0)	52 (17.6)	
45-49	84 (19.7)	26 (21.3)	58 (19.7)	
50-54	61 (14.3)	10 (8.2)	42 (14.2)	
55-60	29 (6.8)	7 (5.7)	22 (7.5)	
> 60	8 (1.9)	1 (0.8)	7 (2.4)	
Mean (SD)	41.7 (9.8)	39.9 (10.0)	42.5 (9.6)	
Sex				0.733**
Female	135 (31.7)	40 (30.5)	95 (32.2)	
Male	291 (68.3)	91 (69.5)	200 (67.8)	
Body weight (kilograms)				0.560*
< 50	4 (0.9)	1 (0.8)	3 (1.0)	
50-59	63 (14.8)	17 (13.0)	46 (15.6)	
60-69	90 (21.1)	33 (25.2)	57 (19.3)	
70-79	112 (26.3)	34 (26.0)	78 (26.4)	
80-89	98 (23.0)	30 (22.9)	68 (23.1)	
90-100	47 (11.0)	13 (9.9)	34 (11.5)	
> 100	12 (2.8)	3 (2.3)	9 (3.1)	
Mean (SD)	74.2 (13.4)	72.6 (14.0)	74.9 (13.2)	
Body height (meters)				0.625*
1.50-1.59	26 (6.1)	5 (3.8)	21 (7.1)	
1.60-1.69	97 (22.8)	34 (26.0)	63 (21.4)	
1.70-1.79	177 (41.5)	52 (39.7)	125 (42.4)	
1.80-1.90	118 (27.7)	37 (28.2)	81 (27.5)	
> 1.90	8 (1.9)	3 (2.3)	5 (1.7)	
Mean (SD)	1.71 (0.1)	1.70 (0.1)	1.72 (0.1)	
Body mass index (kilograms/meters^2)				0.080*
17-19	22 (5.2)	9 (6.9)	13 (4.4)	
20-22	126 (29.6)	39 (29.8)	87 (29.5)	
23-25	158 (37.1)	53 (40.5)	105 (35.6)	
26-28	86 (20.2)	20 (15.3)	66 (22.4)	
29-32	27 (6.3)	7 (5.3)	20 (6.8)	
> 32	7 (1.6)	3 (2.3)	4 (1.4)	
Mean (SD)	24.5 (3.2)	24.1 (3.2)	24.6 (3.1)	
Profession physical activity level				0.098*
Sedentary	121 (28.4)	28 (21.4)	93 (31.5)	
Sitting and walking, without physical efforts	167 (39.2)	59 (45.0)	108 (36.6)	
Sitting and walking, with moderate physical efforts	51 (12.0)	14 (10.7)	37 (12.5)	
Sitting and walking, with heavy physical efforts	3 (0.7)	0 (0.0)	3 (1.0)	
Standing and walking, without physical efforts	20 (4.7)	6 (4.6)	14 (4.7)	
Standing and walking, with moderate physical efforts	54 (12.7)	19 (14.5)	35 (11.9)	
Standing and walking, with heavy physical efforts	10 (2.3)	5 (3.8)	5 (1.7)	
Sport or physical activity background				0.796**
Yes	398 (93.4)	123 (93.9)	275 (93.2)	
No	28 (6.6)	8 (6.1)	20 (6.8)	
Years of sport or physical activity background				0.928*

< 6 months	7 (1.8)	2 (1.6)	5 (1.8)	
6 months-1 year	26 (6.5)	10 (8.1)	16 (5.8)	
2-5 years	56 (14.1)	15 (12.2)	41 (14.9)	
6-10 years	56 (14.1)	17 (13.8)	39 (14.2)	
> 10 years	253 (63.6)	79 (64.2)	174 (63.3)	
Sport or physical activity background weekly practice (minutes)				0.231*
60-119	34 (8.5)	10 (8.1)	24 (8.7)	
120-239	149 (37.4)	43 (35.0)	106 (38.5)	
240-359	93 (23.4)	31 (25.2)	62 (22.5)	
360-479	58 (14.6)	16 (13.0)	42 (15.3)	
480-600	43 (10.8)	14 (11.4)	29 (61.7)	
> 600	21 (5.3)	9 (7.3)	12 (4.4)	
Mean (SD)	293 (221)	317 (255)	283 (203)	
Sport or physical activity background practiced				0.199**
Basketball	18 (4.5)	5 (4.1)	13 (4.7)	
Crossfit	6 (1.5)	0 (0.0)	6 (2.2)	
Cycling	8 (2.0)	0 (0.0)	8 (2.9)	
Futsal	15 (3.8)	4 (3.3)	11 (4.0)	
Gymnastics	5 (1.3)	2 (1.6)	3 (1.1)	
Handball	18 (4.5)	5 (4.1)	13 (4.7)	
Hockey (Roller)	5 (1.3)	5 (4.1)	0 (0.0)	
Mountain Biking	9 (2.3)	4 (3.3)	5 (1.8)	
Running	14 (3.5)	3 (2.4)	11 (4.0)	
Soccer	66 (16.6)	25 (20.3)	41 (14.9)	
Squash	5 (1.3)	0 (0.0)	5 (1.8)	
Swimming	8 (2.0)	4 (3.3)	4 (1.5)	
Tennis	66 (16.6)	16 (13.0)	50 (18.2)	
Volleyball	25 (6.3)	8 (6.5)	17 (6.2)	
Weightlifting/Gym	59 (14.8)	17 (13.8)	42 (15.3)	
Other	31 (7.6)	6 (4.9)	25 (9.1)	
More than one	40 (10.1)	19 (15.4)	21 (7.6)	
Sport or physical activity currently practiced besides Padel				0.915**
Yes	239 (56.1)	74 (56.5)	165 (55.9)	
No	187 (43.9)	57 (43.5)	130 (44.1)	
Years of Sport or physical activity currently practiced besides Padel				0.144*
< 6 months	17 (7.1)	7 (9.5)	10 (6.1)	
6 months-1 year	21 (8.8)	22 (29.7)	50 (30.3)	
2-5 years	72 (30.1)	8 (10.8)	13 (7.9)	
6-10 years	34 (14.2)	13 (17.6)	21 (12.7)	
> 10 years	95 (39.7)	24 (32.4)	71 (43.0)	
Sport or physical activity weekly training (minutes) currently practiced besides Padel				0.308*
< 60	6 (2.5)	2 (2.7)	4 (2.4)	
60-119	55 (23.0)	23 (31.1)	32 (19.4)	
120-239	123 (51.5)	33 (44.6)	90 (54.5)	
240-359	41 (17.2)	12 (16.2)	29 (17.6)	
360-479	11 (4.6)	3 (4.1)	8 (4.8)	
480-600	2 (0.8)	1 (1.4)	1 (0.6)	
> 600	1 (0.4)	0 (0.0)	1 (0.6)	
Mean (SD)	165 (98.8)	157 (96.5)	169 (99.9)	
Sport or physical activity currently practiced besides Padel				0.329**
Crossfit	7 (2.9)	2 (2.7)	5 (3.0)	
Cycling	7 (2.9)	2 (2.7)	5 (3.0)	
Futsal	4 (1.7)	4 (5.4)	0 (0.0)	
Mountain Biking	4 (1.7)	3 (4.1)	1 (0.6)	

Pilates	11 (4.6)	4 (5.4)	7 (4.2)	
Running	23 (9.6)	8 (10.8)	15 (9.1)	
Soccer	9 (3.8)	2 (2.7)	7 (4.2)	
Swimming	5 (2.1)	2 (2.7)	3 (1.8)	
Tennis	15 (6.3)	5 (6.8)	10 (6.1)	
Track and field	3 (1.3)	1 (1.4)	2 (1.2)	
Weightlifting/Gym	128 (53.6)	35 (47.3)	93 (56.4)	
Yoga	3 (1.3)	1 (1.4)	2 (1.2)	
Other	15 (6.1)	4 (5.4)	11 (6.7)	
More than one	5 (2.1)	1 (1.4)	4 (2.4)	
Padel weekly training				0.055*
1	67 (15.7)	23 (17.6)	44 (14.9)	
2	131 (30.8)	48 (36.6)	83 (28.1)	
3	133 (31.2)	36 (27.5)	97 (32.9)	
4	59 (13.8)	15 (11.5)	44 (14.9)	
5	27 (6.3)	7 (5.3)	20 (6.8)	
> 5	9 (2.1)	2 (1.5)	7 (2.4)	
Mean (SD)	2.7 (1.3)	2.6 (1.3)	2.8 (1.3)	
Padel weekly training (minutes)				0.545*
< 60	2 (0.5)	2 (1.5)	0 (0.0)	
60-119	365 (85.7)	109 (83.2)	256 (86.8)	
120-179	32 (7.5)	13 (9.9)	19 (6.4)	
180-239	13 (3.1)	4 (3.1)	9 (3.1)	
240-300	9 (2.1)	3 (2.3)	6 (2.0)	
> 300	5 (1.2)	0 (0.0)	5 (1.7)	
Mean (SD)	96.1 (50.5)	91.8 (34.1)	98.1 (56.2)	
Padel training national localization				0.659**
Açores	3 (0.7)	1 (0.8)	2 (0.7)	
Aveiro	14 (3.3)	5 (3.8)	9 (3.0)	
Beja	5 (1.2)	1 (0.8)	4 (1.3)	
Braga	37 (8.7)	15 (11.5)	22 (7.4)	
Castelo Branco	1 (0.2)	1 (0.8)	0 (0.0)	
Coimbra	12 (2.8)	3 (2.3)	9 (3.0)	
Évora	3 (0.7)	2 (1.5)	1 (0.3)	
Faro	42 (9.9)	11 (8.4)	31 (10.5)	
Leiria	21 (4.9)	8 (6.1)	13 (4.4)	
Lisboa	146 (34.3)	40 (30.5)	106 (35.9)	
Madeira	10 (2.3)	3 (2.3)	7 (2.4)	
Portalegre	5 (1.2)	3 (2.3)	2 (0.7)	
Porto	74 (17.4)	21 (16.0)	53 (17.9)	
Santarém	12 (2.8)	5 (3.8)	7 (2.4)	
Setúbal	30 (7.0)	9 (6.9)	21 (7.1)	
Viana do Castelo	3 (0.7)	1 (0.8)	2 (0.7)	
Vila Real	1 (0.2)	1 (0.8)	0 (0.0)	
Viseu	7 (1.6)	1 (0.8)	6 (2.0)	
Years practicing Padel				**p<0.001***
< 1	3 (0.7)	1 (0.8)	2 (0.7)	
1-3	215 (50.5)	81 (61.8)	134 (45.4)	
4-6	143 (33.6)	39 (29.8)	104 (35.3)	
7-9	38 (8.9)	4 (3.1)	34 (11.5)	
10-12	18 (4.2)	4 (3.1)	14 (4.7)	
13-15	5 (1.2)	1 (0.8)	4 (1.4)	
> 15	4 (0.9)	1 (0.8)	3 (1.0)	
Mean (SD)	4.2 (3.1)	3.6 (2.8)	4.5 (3.2)	
Padel hand dominance				0.654**
Right	401 (94.1)	125 (95.4)	276 (93.5)	
Left	21 (5.6)	6 (4.6)	18 (6.1)	
Do not know	1 (0.2)	0 (0.0)	1 (0.3)	
Padel field side dominance				0.982**

Right	209 (49.1)	65 (49.6)	144 (48.8)	
Left	214 (50.2)	65 (49.6)	149 (50.5)	
Do not know	3 (0.7)	1 (0.8)	2 (0.7)	
Used footwear				0.675**
Padel	387 (90.8)	121 (92.4)	266 (90.2)	
Running	2 (0.5)	0 (0.0)	2 (0.7)	
Squash	1 (0.2)	0 (0.0)	1 (0.3)	
Tennis	36 (8.5)	10 (7.6)	26 (8.8)	
Padel racket shape				0.076**
Diamond	107 (25.1)	30 (22.9)	77 (26.1)	
Teardrop	181 (42.5)	67 (51.1)	114 (38.6)	
Round	122 (28.6)	31 (23.7)	91 (30.8)	
Hybrid	7 (1.6)	0 (0.0)	7 (2.4)	
Do not know	9 (2.1)	3 (2.3)	6 (2.0)	
Racket weight				0.078*
Light (< 370 g)	217 (50.9)	58 (44.3)	159 (53.9)	
Moderate (370-385 g)	183 (43.0)	68 (51.9)	115 (39.0)	
Heavy (> 385 g)	10 (2.3)	1 (0.8)	9 (3.1)	
Do not know	16 (3.8)	4 (3.1)	12 (4.1)	
Racket outer shell				0.559**
Carbon fiber	325 (76.3)	97 (74.0)	228 (77.3)	
Cork	18 (4.2)	7 (5.3)	11 (3.7)	
Fiber glass	24 (5.6)	7 (5.3)	17 (5.8)	
Mixed (carbon + cork)	2 (0.5)	0 (0.0)	2 (0.7)	
Mixed (carbon + glass)	3 (0.7)	0 (0.0)	3 (1.0)	
Do not know	54 (12.7)	20 (15.3)	34 (11.5)	
Racket core				0.376*
Soft	153 (35.9)	36 (27.5)	117 (39.7)	
Medium	10 (2.3)	3 (2.3)	7 (2.4)	
Hard	154 (36.2)	49 (37.4)	105 (35.6)	
Do not know	109 (25.6)	43 (32.8)	66 (22.4)	
Overgrips				0.961*
0	4 (0.9)	3 (2.3)	1 (0.3)	
1	260 (61.0)	76 (58.0)	184 (62.4)	
2	144 (33.8)	46 (35.1)	98 (33.2)	
3	12 (2.8)	4 (3.0)	8 (2.7)	
4	1 (0.2)	0 (0.0)	1 (0.3)	
Do not know	5 (1.2)	2 (1.5)	3 (1.0)	
Playing surface				0.221**
Artificial grass	156 (36.6)	40 (30.5)	116 (39.3)	
Synthetic material	249 (58.5)	84 (64.1)	165 (55.9)	
Do not know	21 (4.9)	7 (5.3)	14 (4.7)	
Padel competitive level				
Masculine (M)				0.739*
1	5 (1.7)	1 (0.8)	4 (1.3)	
2	32 (11.0)	8 (6.1)	24 (8.1)	
3	102 (34.9)	31 (23.7)	71 (24.1)	
4	108 (37.0)	30 (22.9)	78 (26.4)	
5	40 (13.7)	12 (9.2)	28 (9.5)	
6	5 (1.7)	2 (1.5)	3 (1.0)	
Mean (SD)	3.6 (1.0)	3.6 (1.0)	3.5 (1.0)	
Female (F)				0.329*
1	3 (2.2)	1 (0.8)	2 (0.7)	
2	15 (11.2)	6 (4.6)	9 (3.0)	
3	44 (32.8)	11 (8.4)	33 (11.2)	
4	52 (38.8)	21 (16.0)	31 (10.5)	
5	16 (11.9)	6 (4.6)	10 (3.4)	
6	4 (3.0)	2 (1.5)	2 (0.7)	
Mean (SD)	3.6 (1.0)	3.7 (1.1)	3.5 (1.0)	

Mixed (MX)				0.948*
1	0 (0.0)	0 (0.0)	0 (0.0)	
2	13 (10.2)	5 (3.8)	8 (2.7)	
3	38 (29.7)	13 (9.9)	25 (8.5)	
4	56 (43.8)	22 (16.8)	34 (11.5)	
5	17 (13.3)	7 (5.3)	10 (3.4)	
6	4 (3.1)	0 (0.0)	4 (1.3)	
Mean (SD)	3.7 (0.9)	3.7 (0.9)	3.7 (1.0)	
Padel monthly competitions				0.462*
0	10 (2.3)	2 (1.5)	8 (2.7)	
1-3	193 (45.3)	59 (45.0)	134 (45.4)	
4-6	112 (26.3)	35 (26.7)	77 (26.1)	
7-9	40 (9.4)	11 (8.4)	29 (9.8)	
10-12	49 (11.5)	15 (11.5)	34 (11.5)	
13-15	8 (1.9)	5 (3.8)	3 (1.0)	
> 15	14 (3.3)	4 (3.1)	10 (3.4)	
Mean (SD)	4.9 (4.3)	5.2 (4.4)	4.8 (4.2)	
Warm-up before training/competitions?				0.097*
No	84 (19.7)	29 (22.1)	55 (18.6)	
Yes, < 10 minutes	283 (66.4)	90 (68.7)	193 (65.4)	
Yes, 10-20 minutes	55 (12.9)	11 (8.4)	44 (14.9)	
Yes, 21-30 minutes	3 (0.7)	1 (0.8)	2 (0.7)	
Yes, > 30 minutes	1 (0.2)	0 (0.0)	1 (0.3)	
Warm-up exercises used				
Balance exercises	23 (2.6)	4 (1.6)	19 (3.0)	0.153**
Core exercises	32 (3.6)	9 (3.5)	23 (3.6)	0.738**
Dynamic stretching	134 (15.0)	36 (14.2)	98 (15.3)	0.239**
Jogging	31 (3.5)	5 (2.0)	26 (4.1)	0.067**
Mobility exercises	280 (31.3)	86 (33.9)	194 (30.3)	0.982**
Plyometrics	44 (4.9)	11 (4.3)	33 (5.1)	0.383**
Running drills	112 (12.5)	37 (14.6)	75 (11.7)	0.542**
Self-massage	16 (1.8)	2 (0.8)	14 (2.2)	0.107**
Sport-specific exercises	50 (5.6)	16 (6.3)	34 (5.3)	0.839**
Sprint	15 (1.7)	5 (2.0)	10 (1.6)	0.825**
Static stretching	121 (13.5)	33 (13.0)	88 (13.7)	0.327**
Strengthening exercises	37 (4.1)	10 (3.9)	27 (4.2)	0.607**
Reasons to perform warm-up				0.457**
Concentration	10 (2.9)	4 (3.9)	6 (2.5)	
Fitness/Performance	12 (3.5)	6 (5.9)	6 (2.5)	
Injury prevention	281 (82.2)	79 (77.5)	202 (84.2)	
Nervousness reduction	4 (1.2)	2 (2.0)	2 (0.8)	
Routine	35 (10.2)	11 (10.8)	24 (10.0)	
Cool-down after training/competitions?				0.271*
No	209 (49.1)	68 (51.9)	141 (47.8)	
Yes, < 10 minutes	189 (44.4)	58 (44.3)	131 (44.4)	
Yes, 10-20 minutes	21 (4.9)	5 (3.8)	16 (5.4)	
Yes, 21-30 minutes	3 (0.7)	0 (0.0)	3 (1.0)	
Yes, > 30 minutes	4 (0.9)	0 (0.0)	4 (1.4)	
Cool-down strategies used				0.534**
Active recovery	11 (5.1)	6 (9.5)	5 (3.2)	
Cryotherapy	5 (2.3)	0 (0.0)	5 (3.2)	
Electric stimulation	4 (1.8)	2 (3.2)	2 (1.3)	
Foam roller	5 (2.3)	1 (1.6)	4 (2.6)	
Manual massage	6 (2.8)	1 (1.6)	5 (3.2)	
Massage guns	8 (3.7)	3 (4.8)	5 (3.2)	
Nutrition/Supplementation	9 (4.1)	3 (4.8)	6 (3.9)	
Passive recovery/Rest	2 (0.9)	0 (0.0)	2 (1.3)	
Sauna	1 (0.5)	0 (0.0)	1 (0.6)	
Stretching	166 (76.5)	47 (74.6)	119 (77.3)	

Reasons to perform cool-down				0.583**
Injury prevention	111 (51.2)	30 (47.6)	81 (52.6)	
Fatigue reduction	44 (20.3)	11 (17.5)	33 (21.4)	
Relaxation	25 (11.5)	10 (15.9)	15 (9.7)	
Routine	10 (4.6)	2 (3.2)	8 (5.2)	
Well-being and comfort	27 (12.4)	10 (15.9)	17 (11.0)	
Existence and monitoring by a Padel instructor				0.892**
No	65 (15.3)	19 (14.5)	46 (15.6)	
Yes and non-monitored	72 (16.9)	21 (16.0)	51 (17.3)	
Yes and monitored	289 (67.8)	91 (69.5)	198 (67.1)	
Monitored by a health professional				0.065**
No	285 (62.2)	90 (68.7)	175 (59.3)	
Yes	161 (37.8)	41 (31.3)	120 (40.7)	

Note: * Mann-Whitney U; **chi-square test; bold – significant statistical difference

Table 2. Exercises/drills included in the warm-up sessions (n=342).

Self-massage	Core Exercises	Jumping / Plyometrics	Sport-specific Exercises	Static Stretching	Mobility Exercises	Dynamic Stretching	Running Drills	Jogging	Strength Exercises	Sprint	Balance Exercises	n (%)
		✓		✓	✓	✓		✓		✓		60 (17.
					✓	✓						20 (5.8
					✓		✓					19 (5.6
					✓							17 (5.0
				✓	✓	✓	✓		✓			14 (4.1
			✓	✓	✓		✓				✓	12 (3.5
				✓	✓	✓						11 (3.2
			✓		✓							8 (2.3)
			✓		✓	✓						6 (1.8)
			✓									6 (1.8)
			✓		✓	✓						5 (1.5)
	✓				✓							5 (1.5)
						✓						4 (1.2)
						✓	✓	✓				4 (1.2)
		✓	✓	✓	✓	✓	✓	✓	✓			4 (1.2)
				✓			✓					4 (1.2)
✓					✓	✓	✓					4 (1.2)

Note: From the different 122 warm-up variations 105 (86%) were chosen below 1%.

Table 3. Padel practitioner's reported injuries characterization (n=295).

**Variable**	**n (%)**
Total nº of injuries	
1	101 (34.2)
2	117 (39.7)
3	51 (17.3)
4	15 (5.1)
5	5 (1.7)
> 5	6 (2.0)
Mean total sample (SD)	0.9 (1.4)
Mean per injured athlete (SD)	2.1 (1.2)
Nº of injuries in the last 12 months	
0	62 (21.0)
1	148 (50.2)
2	69 (23.4)
3	13 (4.4)
4	2 (0.7)
5	1 (0.3)
Mean total sample (SD)	0.8 (0.9)
Mean per injured athlete (SD)	1.5 (0.7)
Incidence (1000 h)	
Mean total sample (SD)	3.4 (4.7)
Mean per injured athlete (SD)	6.1 (4.9)
Injury localization	
Head	1 (0.3)
Face	2 (0.7)
Neck	3 (1.0)
Cervical Spine	2 (0.7)
Thoracic Spine	1 (0.3)
Middle Back	1 (0.3)
Abdominal	2 (0.7)
Lower Back	5 (1.7)
Lumbar Spine	5 (1.7)
Shoulder	31 (10.5)
Upper Arm (anterior)	5 (1.7)
Upper Arm (posterior)	2 (0.7)
Elbow	53 (18.0)
Forearm (anterior)	3 (1.0)
Forearm (posterior)	3 (1.0)
Wrist	14 (4.7)
Hand/Fingers	5 (1.7)
Hip	1 (0.3)
Groin	2 (0.7)
Thigh (anterior)	4 (1.4)
Thigh (posterior)	4 (1.4)
Knee	37 (12.5)
Lower Leg (anterior)	19 (6.4)
Lower Leg (posterior)	30 (10.2)
Ankle	49 (16.6)
Foot/Fingers	11 (3.5)
Injury type	
Bone Injury	8 (2.7)
Bursitis	2 (0.7)
Cartilage Injury	1 (0.3)
Concussion	1 (0.3)
Discal Injury	4 (1.4)
Eye Injury	1 (0.3)
Fascial Injury	11 (3.7)
Joint Injury	35 (11.9)

Laceration/Abrasion/Bleeding	3 (1.0)
Ligament Injury	34 (11.5)
Meniscal Injury	6 (2.0)
Muscular Injury	77 (26.1)
Pain	7 (2.4)
Tendon Injury	105 (35.6)
Injury occurrence situation	
Warm-up (training)	4 (1.4)
Warm-up (competition)	5 (1.7)
During training	97 (32.9)
During competition (1st set)	50 (17.0)
During competition (2nd set)	58 (19.7)
During competition (3rd set)	26 (8.8)
During competition (> 3rd set)	16 (5.4)
Cool-down (training)	7 (2.4)
Cool-down (competition)	8 (2.7)
Overuse	24 (8.1)
Perceived injury reason	
Actions performed in a cold environment	1 (0.3)
Backward movement/displacement	12 (4.1)
Forward movement/displacement	37 (12.5)
Sideways movement/displacement	27 (9.2)
Incorrect technical action in bandeja	16 (5.4)
Incorrect technical action in chiquita	1 (0.3)
Incorrect technical action in drop shot	3 (1.0)
Incorrect technical action in lob	1 (0.3)
Incorrect technical action in power shot	5 (1.7)
Incorrect technical action in smash	9 (3.1)
Incorrect technical action in vibora	4 (1.7)
Incorrect technical action in volley	5 (1.7)
Contact with another player	2 (0.7)
Contact with back glass	6 (2.0)
Contact with lateral glass	7 (2.4)
Contact with ball	11 (3.7)
Contact with net	1 (0.3)
Contact with post	1 (0.3)
Contact with racket	6 (2.0)
Slip on ball	3 (1.0)
Inadequate footwear	3 (1.0)
Inadequate grip	1 (0.3)
Inadequate racket	14 (4.7)
Inappropriate infrastructure/court	6 (2.0)
Insufficient warm-up	1 (0.3)
Lack of physical preparation	2 (0.7)
Previous clinical condition	1 (0.3)
Fatigue/DOMS/Overload	71 (24.1)
Unknown	38 (12.9)
Return-to-sport duration	
< 1 week	30 (10.2)
1-2 weeks	55 (18.6)
3-4 weeks	68 (23.1)
1-3 months	85 (28.8)
4-6 months	31 (10.5)
7-12 months	17 (5.8)
> 1 year	9 (3.1)
Injury history	
Recurrence	81 (27.5)
First time	214 (72.5)
Injury management	

No intervention	6 (2.0)
Active self-management	18 (6.1)
Self-Medication/Supplementation	8 (2.7)
Physiotherapist	142 (48.1)
Physician (surgery)	26 (8.8)
Physician (injection)	16 (5.4)
Physician (medication)	34 (11.5)
Non-conventional medicine	16 (5.4)
Rest	29 (9.8)

Table 4. Injury type and localization distribution (n=295).

***Injury type*; localization**	**n (%)**
Bursitis	
Shoulder	2 (100.0)
Cartilage	
Knee	1 (100.0)
Concussion	
Head	1 (100.0)
Discal	
Cervical Spine	2 (50.0)
Lumbar Spine	2 (50.0)
Eye	
Face	1 (100.0)
Laceration/Abrasion/Bleeding	
Face	1 (33.3)
Knee	1 (33.3)
Upper arm (anterior)	1 (33.3)
Joint	
Ankle	23 (65.7)
Elbow	4 (11.4)
Hand/Fingers	1 (2.7)
Knee	3 (8.6)
Shoulder	4 (11.4)
Fascial	
Foot/Fingers	7 (63.6)
Knee	2 (18.2)
Thigh (anterior)	2 (18.2)
Ligament	
Ankle	12 (35.3)
Elbow	3 (8.8)
Hand/Fingers	1 (2.9)
Knee	13 (38.2)
Shoulder	2 (5.9)
Wrist	3 (8.8)
Meniscal	
Knee	6 (100.0)
Muscular	
Abdominal	1 (1.5)
Ankle	2 (2.9)
Foot/Fingers	1 (1.5)
Forearm (anterior)	1 (1.5)
Groin	1 (1.5)
Hand/Fingers	1 (1.5)
Hip	1 (1.5)
Leg (anterior)	18 (26.5)
Leg (posterior)	27 (39.7)
Lower Back	3 (4.4)
Lumbar Spine	2 (2.9)
Middle Back	1 (1.5)
Neck	2 (2.9)
Shoulder	1 (1.5)
Thigh (anterior)	2 (2.9)
Thigh (posterior)	3 (4.4)
Thoracic Spine	1 (1.5)
Bone	
Ankle	2 (25.0)
Foot/Fingers	2 (25.0)
Hand/Fingers	2 (25.0)

Wrist	2 (25.0)
Tendon	
Ankle	10 (9.5)
Elbow	46 (43.8)
Foot/Fingers	1 (1.0)
Forearm (anterior)	2 (1.9)
Forearm (posterior)	3 (2.9)
Groin	1 (1.0)
Knee	9 (8.6)
Lower Back	1 (1.0)
Lower Leg (anterior)	1 (1.0)
Lower Leg (posterior)	3 (2.9)
Neck	1 (1.0)
Shoulder	13 (12.4)
Thigh (posterior)	1 (1.0)
Upper Arm (anterior)	3 (2.9)
Upper Arm (posterior)	2 (1.9)
Wrist	8 (7.6)
Pain	
Abdominal	1 (14.3)
Knee	2 (28.6)
Lower Back	1 (14.3)
Lumbar Spine	1 (14.3)
Upper Arm (anterior)	1 (14.3)
Wrist	1 (14.3)

Table 5a. Padel practitioner's variables by injury type and localization (n=295).

Injury Localization	Perceived reason																												
	Cold environment	*Backward movement*	*Forward movement*	*Sideways movement*	*Bandeja*	*Chiquita*	*Drop Shot*	*Lob*	*Power Shot*	*Smash*	*Vibora*	*Volley*	*Player contact*	*Back glass contact*	*Lateral glass contact*	*Ball contact*	*Net contact*	*Post contact*	*Racket contact*	*Slip on ball*	*Footwear*	*Grip*	*Racket*	*Infrastructure / court*	*Insufficient warm-up*	*Poor physical conditioning*	*Clinical condition*	*Fatigue*	*Unknown*
Head	-	-	-	-	-	-	-	-	-	-	-	-	-	-	-	-	-	-	1	-	-	-	-	-	-	-	-	-	-
Face	-	-	-	-	-	-	-	-	-	-	-	-	-	1	-	1	-	-	-	-	-	-	-	-	-	-	-	-	-
Neck	-	-	-	-	-	-	-	-	-	-	-	-	-	-	-	-	-	-	-	-	-	-	1	-	-	-	-	-	2
Cervical Spine	-	-	-	-	-	-	-	-	-	-	-	-	-	-	-	-	-	-	-	-	-	-	-	-	-	-	-	1	1
Thoracic Spine	-	-	-	-	-	-	-	-	-	-	-	-	-	-	-	-	-	-	-	-	-	-	-	1	-	-	-	-	-
Middle Back	-	-	-	-	-	-	-	-	1	-	-	-	-	-	-	-	-	-	-	-	-	-	-	-	-	-	-	-	-
Abdominal	-	-	-	-	-	-	-	-	-	-	-	-	-	-	-	-	-	-	-	-	-	-	-	-	1	-	-	1	-
Lower Back	-	-	1	-	1	-	-	-	-	-	-	-	-	-	-	1	-	-	-	-	-	-	-	-	-	-	-	1	1
Lumbar Spine	-	-	-	-	-	-	-	-	-	-	-	-	-	-	-	1	-	-	-	-	1	-	-	-	-	-	-	1	2
Shoulder	-	-	-	-	5	-	-	-	2	3	-	-	-	1	3	1	-	-	-	-	-	1	2	-	-	1	-	7	5
Upper Arm (anterior)	-	-	-	-	1	-	-	-	-	-	-	-	-	-	-	-	-	-	-	-	-	-	-	-	-	-	-	2	1
Upper Arm (posterior	-	-	-	-	1	-	-	-	-	-	-	-	-	-	-	-	-	-	-	-	-	-	1	-	-	-	-	-	-
Elbow	-	-	-	1	3	1	2	-	1	5	2	3	-	-	1	3	-	-	3	-	-	-	9	-	-	-	1	13	5
Forearm (anterior)	-	-	-	-	-	-	-	-	-	-	-	-	-	-	-	-	-	-	-	-	-	-	-	-	-	-	-	1	2
Forearm (posterior)	-	-	-	-	-	-	-	-	-	-	1	-	-	-	-	-	-	-	-	-	-	-	-	-	-	-	-	2	-
Wrist	-	2	-	-	-	-	-	1	1	-	-	-	-	-	2	-	-	-	2	-	-	-	1	-	-	-	-	3	2
Hand/Fingers	-	1	-	-	-	-	-	-	-	-	-	-	-	1	1	1	1	-	-	-	-	-	-	-	-	-	-	-	-
Hip	-	1	-	-	-	-	-	-	-	-	-	-	-	-	-	-	-	-	-	-	-	-	-	-	-	-	-	-	-
Groin	-	-	-	1	-	-	-	-	-	-	-	-	-	-	-	-	-	-	-	-	-	-	-	-	-	-	-	1	-
Thigh (anterior)	-	-	3	-	-	-	-	-	-	-	-	-	-	-	-	-	-	-	-	-	-	-	-	1	-	-	-	-	-
Thigh (posterior)	-	-	1	-	-	-	-	-	-	-	-	-	-	-	-	-	-	-	-	-	-	-	-	1	-	-	-	2	-
Knee	-	2	3	9	-	-	-	-	-	-	-	1	1	-	-	-	-	1	-	1	-	-	-	1	-	1	-	9	8
Lower Leg (anterior)	1	1	8	1	-	-	-	-	-	-	-	-	-	-	-	-	-	-	-	-	-	-	-	-	-	-	-	6	2
Lower Leg (posterior)	-	2	12	1	-	-	-	-	-	-	-	-	-	-	-	1	-	-	-	-	-	-	-	-	-	-	-	11	3
Ankle	-	3	5	13	5	-	1	-	-	-	1	1	1	-	2	2	-	-	-	2	2	-	-	2	-	-	-	7	2
Foot/Fingers	-	-	3	1	1	-	-	-	-	-	-	-	-	1	-	-	-	-	-	-	-	-	-	-	-	-	-	3	2
Injury Type																													
Bone Injury	-	3	-	-	1	-	-	-	-	-	-	-	-	1	1	1	-	-	-	-	1	-	-	-	-	-	-	-	-
Bursitis	-	-	-	-	-	-	-	-	-	1	-	-	-	-	-	-	-	-	-	-	-	-	-	-	-	-	-	-	1
Cartilage Injury	-	-	1	-	-	-	-	-	-	-	-	-	-	-	-	-	-	-	-	-	-	-	-	-	-	-	-	-	-

Concussion	-	-	-	-	-	-	-	-	-	-	-	-	-	-	-	-	-	-	1	-	-	-	-	-	-	-	-	-	-
Discal Injury	-	-	-	-	-	-	-	-	-	-	-	-	-	-	-	-	-	-	-	-	-	-	-	-	-	-	-	2	2
Eye Injury	-	-	-	-	-	-	-	-	-	-	-	-	-	-	-	1	-	-	-	-	-	-	-	-	-	-	-	-	-
Fascial Injury	-	-	-	1	-	-	-	-	-	-	-	-	-	-	-	-	-	-	-	-	-	-	-	-	-	-	-	2	3
Joint Injury	-	2	1	11	2	-	-	-	-	-	1	2	-	1	4	1	-	-	1	1	1	-	-	2	-	-	-	4	1
Laceration	-	-	1	-	-	-	-	-	-	-	-	-	-	1	-	-	-	-	-	-	-	-	-	-	-	-	-	1	-
Ligament Injury	-	2	4	6	2	-	-	-	-	3	-	1	1	2	-	1	-	-	1	-	-	-	-	-	-	1	1	2	7
Meniscal Injury	-	1	-	2	-	-	-	-	-	-	-	-	-	-	-	-	-	-	-	-	-	-	-	-	-	-	-	2	1
Muscular Injury	1	4	22	1	5	-	-	-	1	-	-	-	-	-	1	2	1	-	-	1	-	1	-	3	1	-	-	22	11
Pain	-	-	-	-	-	-	-	-	-	1	-	-	-	-	-	-	-	-	-	1	1	-	-	-	-	-	-	3	1
Tendon Injury	-	-	3	6	6	1	3	1	4	4	3	2	1	1	1	5	-	1	3	-	-	-	14	1	-	1	-	33	11

Note: ☐ 0% to ≤25%; ☐ >25% to ≤50%; ☐ >50% to ≤75%; ☐ >75% to 100%

Table 5b. Padel practitioner's variables by injury type and localization (n=295). 16

Injury Localization	Situation										Court Zone																	
	Warm-up (training)	*Warm-up (competition)*	*During training*	*During competition (1st set)*	*During competition (2nd set)*	*During competition (3rd set)*	*During competition (+3rd set)*	*Cool-down (training)*	*Cool-down (competition)*	*Overuse*	*1*	*2*	*3*	*4*	*5*	*6*	*7*	*8*	*9*	*10*	*11*	*12*	*13*	*14*	*15*	*16*	*17*	*18*
Head	-	-	-	-	1	-	-	-	-	-	1	-	-	-	-	-	-	-	-	-	-	-	-	-	-	-	-	-
Face	-	-	-	1	-	1	-	-	-	-	-	-	-	-	-	1	-	-	-	1	-	-	-	-	-	-	-	-
Neck	-	-	1	-	1	-	-	-	-	1	1	1	-	-	-	-	-	-	-	-	-	-	-	-	-	-	1	-
Cervical Spine	-	-	-	-	-	-	-	1	-	1	-	-	-	-	-	-	-	2	-	-	-	-	-	-	-	-	-	-
Thoracic Spine	-	-	-	-	1	-	-	-	-	-	-	-	-	-	-	-	-	-	1	-	-	-	-	-	-	-	-	-
Middle Back	-	-	-	-	1	-	-	-	-	-	-	-	-	-	-	-	-	-	-	1	-	-	-	-	-	-	-	-
Abdominal	-	-	-	1	1	-	-	-	-	-	-	-	-	-	-	-	-	-	-	-	1	-	-	-	-	-	1	-
Lower Back	-	-	3	-	-	2	-	-	-	-	-	-	-	1	-	-	-	-	-	1	1	-	-	2	-	-	-	-
Lumbar Spine	-	1	3	-	-	-	-	-	-	1	-	1	-	1	-	1	-	1	-	-	-	-	-	1	-	-	-	-
Shoulder	1	-	10	5	2	6	1	2	-	4	1	-	2	1	3	4	-	3	1	4	4	1	-	4	1	1	1	-
Upper Arm (anterior)	1	-	3	-	1	-	-	-	-	-	1	1	-	-	-	-	-	-	1	-	1	-	-	-	-	-	1	-
Upper Arm (posterior)	-	-	1	-	-	-	-	-	-	1	-	-	-	-	1	-	-	1	-	-	-	-	-	-	-	-	-	-
Elbow	1	-	22	2	7	3	2	1	5	10	4	7	2	1	7	2	1	7	1	1	7	1	1	5	1	2	3	-
Forearm (anterior)	-	-	1	-	-	-	1	-	1	-	-	-	-	-	1	-	-	1	-	-	-	-	-	-	1	-	-	-
Forearm (posterior)	-	-	1	-	-	-	2	-	-	-	-	-	-	-	-	-	-	1	-	-	-	-	-	-	-	1	1	-
Wrist	-	-	5	1	3	1	1	-	-	3	1	1	-	3	1	-	1	-	1	1	2	-	-	1	-	1	-	1
Hand/Fingers	-	-	2	3	-	-	-	-	-	-	-	-	-	-	-	1	2	1	-	-	-	-	-	-	-	-	1	-
Hip	-	-	-	-	1	-	-	-	-	-	-	1	-	-	-	-	-	-	-	-	-	-	-	-	-	-	-	-
Groin	-	-	-	1	-	-	1	-	-	-	-	-	-	-	-	-	1	-	-	1	-	-	-	-	-	-	-	-
Thigh (anterior)	-	-	-	2	1	1	-	-	-	-	-	2	-	-	-	-	-	1	-	-	1	-	-	-	-	-	-	-
Thigh (posterior)	-	1	1	-	-	2	-	-	-	-	-	-	-	-	1	-	-	-	-	-	2	-	-	1	-	-	-	-
Knee	-	1	11	4	10	5	3	-	1	2	1	3	1	-	8	4	1	7	2	2	1	-	1	1	-	3	1	1
Lower Leg (anterior)	1	-	2	10	5	-	1	-	-	-	-	2	-	-	4	-	-	4	-	4	2	-	-	-	-	1	1	1
Lower Leg (posterior)	-	2	7	7	8	1	4	1	-	-	-	4	3	-	4	1	1	3	1	2	7	2	1	-	-	-	-	1
Ankle	-	-	23	12	9	4	-	1	-	-	3	10	-	1	2	2	-	6	6	2	9	1	-	2	1	1	1	2
Foot/Fingers	-	-	1	1	6	-	-	1	1	1	-	2	-	1	1	1	1	1	1	-	1	-	-	-	1	-	1	-
Injury Type																												
Bone Injury	-	-	2	4	2	-	-	-	-	-	-	1	-	-	-	-	1	1	2	1	1	-	-	-	-	-	1	-
Bursitis	-	-	-	-	-	2	-	-	-	-	-	-	-	-	-	-	-	-	-	-	-	2	-	-	-	-	-	-
Cartilage Injury	-	-	-	-	1	-	-	-	-	-	-	-	-	-	-	-	-	1	-	-	-	-	-	-	-	-	-	-
Concussion	-	-	-	-	1	-	-	-	-	-	1	-	-	-	-	-	-	-	-	-	-	-	-	-	-	-	-	-
Discal Injury	-	-	1	-	-	-	-	1	-	2	-	-	-	-	-	1	-	3	-	-	-	-	-	-	-	-	-	-
Eye Injury	-	-	-	-	-	1	-	-	-	-	-	-	-	-	-	-	-	-	-	1	-	-	-	-	-	-	-	-
Fascial Injury	-	-	-	2	6	-	-	1	1	1	-	-	-	1	2	1	1	2	-	-	2	-	1	-	-	-	1	-

Joint Injury	1	-	12	8	7	5	1	-	-	1	2	4	1	-	2	4	-	6	2	1	6	1	-	2	1	1	1	1
Laceration	-	-	-	1	1	1	-	-	-	-	-	-	-	-	-	1	-	-	-	-	-	-	-	-	-	1	1	-
Ligament Injury	-	-	15	6	5	6	1	-	1	-	1	4	-	2	5	4	-	5	3	1	3	1	-	1	-	2	1	1
Meniscal Injury	-	-	3	-	1	1	1	-	-	-	-	-	-	-	4	-	-	1	-	1	-	-	-	-	-	-	-	-
Muscular Injury	1	4	19	19	19	5	6	2	-	2	1	10	3	2	12	2	2	8	4	10	12	1	1	3	1	2	2	1
Pain	-	-	5	1	-	1	-	-	-	-	-	-	1	1	-	-	-	-	2	-	-	-	-	2	-	-	1	-
Tendon Injury	2	1	40	9	15	4	7	3	6	18	8	16	3	3	8	4	4	12	2	5	15	2	1	7	3	4	5	3

Note: 0% to ≤25%; >25% to ≤50%; >50% to ≤75%; >75% to 100%

Table 5c. Padel practitioner's variables by injury type and localization (n=295).

Injury Localization	Return-to-sport							Clinical history		Management								
	- 1 week	*1-2 weeks*	*3-4 weeks*	*1-3 months*	*4-6 months*	*7-12 months*	*+ 1 year*	*Recurrence*	*First time*	*No intervention*	*Self-management*	*Self-medication*	*Physiotherapist*	*Physician (surgery)*	*Physician (injection)*	*Physician (medication)*	*Non-conventional medicine*	*Rest*
Head	1	-	-	-	-	-	-	-	1	-	-	1	-	-	-	-	-	-
Face	-	2	-	-	-	-	-	-	2	-	-	-	-	1	-	1	-	-
Neck	-	-	2	-	-	1	-	1	2	-	-	-	2	-	1	-	-	-
Cervical Spine	-	1	-	-	1	-	-	-	2	-	-	-	-	1	-	-	1	-
Thoracic Spine	-	1	-	-	-	-	-	-	1	-	-	1	-	-	-	-	-	-
Middle Back	-	1	-	-	-	-	-	-	1	-	-	-	1	-	-	-	-	-
Abdominal	-	1	-	1	-	-	-	-	2	-	1	-	-	-	-	1	-	-
Lower Back	-	2	3	-	-	-	-	4	1	-	-	-	3	-	1	-	-	1
Lumbar Spine	1	1	1	2	-	-	-	2	3	-	-	-	4	-	-	1	-	-
Shoulder	3	6	7	9	3	3	-	10	21	-	-	1	17	1	4	4	3	1
Upper Arm (anterior)	1	3	-	1	-	-	-	2	3	-	-	-	1	-	-	2	-	2
Upper Arm (posterior)	-	-	-	-	2	-	-	-	2	-	-	-	2	-	-	-	-	-
Elbow	9	7	10	18	4	3	2	16	37	2	7	1	27	-	5	2	5	4
Forearm (anterior)	-	-	1	2	-	-	-	-	3	-	1	-	2	-	-	-	-	-
Forearm (posterior)	1	1	1	-	-	-	-	1	2	-	-	-	1	-	-	1	1	-
Wrist	1	3	3	6	1	-	-	3	11	-	1	3	2	1	1	1	1	4
Hand/Fingers	2	-	1	2	-	-	-	-	5	-	-	-	1	1	1	2	-	-
Hip	-	-	1	-	-	-	-	-	1	-	-	-	1	-	-	-	-	-
Groin	-	-	1	-	1	-	-	-	2	-	-	-	1	-	-	-	-	1
Thigh (anterior)	-	-	1	2	1	-	-	-	4	-	-	-	3	-	-	1	-	-
Thigh (posterior)	-	1	2	1	-	-	-	1	3	-	-	-	3	-	-	-	-	1
Knee	2	4	6	9	7	4	5	11	26	2	-	-	9	12	2	8	1	3
Lower Leg (anterior)	1	2	5	9	2	-	-	3	16	1	1	1	14	1	-	-	-	1
Lower Leg (posterior)	1	6	10	9	2	2	-	8	22	-	3	-	21	-	-	3	1	2
Ankle	5	13	9	11	5	4	2	18	31	1	4	-	20	6	-	6	3	9
Foot/Fingers	2	-	4	3	2	-	-	1	10	-	-	-	7	2	1	1	-	-
Injury Type																		
Bone Injury	-	-	1	3	4	-	-	-	8	-	-	-	-	3	-	4	-	1
Bursitis	-	-	-	-	1	1	-	-	2	-	-	-	1	-	1	-	-	-
Cartilage Injury	-	-	1	-	-	-	-	1	-	-	-	-	-	-	-	1	-	-
Concussion	1	-	-	-	-	-	-	-	1	-	-	1	-	-	-	-	-	-
Discal Injury	-	1	-	2	1	-	-	2	2	-	-	-	1	1	-	1	1	-
Eye Injury	-	1	-	-	-	-	-	-	1	-	-	-	-	-	-	1	-	-
Fascial Injury	2	-	3	3	2	-	1	1	10	1	-	-	7	1	1	-	-	1

Joint Injury	3	9	8	13	1	-	1	17	18	-	2	-	16	-	1	6	3	7
Laceration	-	3	-	-	-	-	-	-	3	-	-	-	-	1	-	2	-	-
Ligament Injury	1	3	3	12	6	5	4	7	27	-	1	12	10	1	5	5	1	1
Meniscal Injury	-	-	1	2	2	1	-	2	4	-	-	-	-	4	-	2	-	-
Muscular Injury	6	16	25	23	4	3	-	20	57	1	5	2	52	1	3	4	4	5
Pain	2	2	2	1	-	-	-	3	4	1	-	-	2	-	-	1	1	2
Tendon Injury	15	20	24	26	10	7	3	28	77	3	10	2	51	5	9	7	6	12

Note: ☐ 0% to ≤25%; ☐ >25% to ≤50%; ☐ >50% to ≤75%; ☐ >75% to 100%

Table 6. Spearman correlations between injury, sport, and sociodemographic variables (n=295).

Variables	BMI	Padel training duration	Padel years	Field side	Racket weight	Racket core	Overgrips	Playing surface	Padel level	Padel competitions	Warm-up	Cool-down	Instructor	Health professional	Padel injuries	1-year Padel injuries
Sex	0.339***	-	-	-	-	0.117*	-	-	-	-	-	-	-	-	-	-
Age	0.192***	-	-	-	-	-	-	-	-	-	-	-	-	-	-	-
BMI		0.148*	0.181**	0.196***	0.247***	0.206***	0.195***	-	-	-	-	-	-0.199***	-	0.117*	-
Work physical activity level	-	-	0.134*	-	-	-	-	-	-	-	-	-	-	-	-	-
Past sport practice	-	0.186**	-	-	-	-	-	-	-	-	-	-	-	-	-	-
Sports engagement + Padel	-	-		-0.132*	-	-	-	-	-0.235*	-	0.165**	-	-	-	-	-
Padel weekly training	-	0.269***	-	-	-	-	-	-	-	0.149*	-	-	-	0.114*	-	-
Padel training duration	-	-	-	-	-	-	-	-	-	-	-	-	-	-	-	0.156**
Padel years	-	-	-	-	-	-	-	-0.216***	-	-	-	-	-0.229***	-	0.140*	-0.235***
Dominant hand	-	-	-	-0.233***	-	-	-	-	-	-	-	-	-	-	-	-
Racket weight	-	-	-	-	-	0.166**	0.158**	-	-	-	-	-	-	-	-	-
Racket core	-	-	-	-	-	-	-	-	-	0.132*	0.134*	-	-	-	-	-
Overgrips	-	-	-	-	-	-	-	-	-	-	-	-	-	-	-	0.119*
Padel Level	-	-	-	-	-	-	-	-	-	-0.230*	-	-	-	-	-	-
Warm-up	-	-	-	-	-	-	-	-	-	-	-	0.290***	-	0.120*	-	-
Padel injuries	-	-	-	-	-	-	-	-	-	-	-	-	-	-	-	0.448***

Note: Only the significant statistically correlations are displayed; *** p≤0.001; ** p≤0.01; *p≤0.05

Table 7. Logistic regressions between the sociodemographic, sport, and injury variables (n=295).

Injury	Factor - Level	*β* (95% CI)	*SE*	*z*	Odds Ratio (95% CI)	*P*	*R*²	*χ*²	AUC
Tendon						*0.012*	*0.029*	*6.34*	*0.567*
	Padel training duration	-0.008 [-0.015; -0.000]	0.004	-2.05	0.992 [0.985; 1.000]	0.041			
Ligament						*0.028*	*0.111*	*17.20*	*0.711*
	Work physical activity level								
	Sitting and walking, without physical efforts	0.464 [-0.514; 1.440]	0.049	0.93	1.590 [0.598; 4.224]	0.353			
	Sitting and walking, with moderate physical efforts	0.359 [-0.952; 1.670]	0.669	0.54	1.432 [0.386; 5.309]	0.592			
	Sitting and walking, with heavy physical efforts	1.487 [-1.047; 4.020]	1.293	1.15	4.424 [0.351; 55.764]	0.250			
	Standing and walking, without physical efforts	0.023 [-2.180; 2.230]	1.124	0.02	1.024 [0.113; 9.265]	0.983			
	Standing and walking, with moderate physical efforts	1.254 [0.035; 2.470]	0.622	2.03	3.506 [1.036; 11.864]	0.044			
	Standing and walking, with heavy physical efforts	2.562 [0.479; 4.650]	1.063	2.41	12.965 [1.615; 104.096]	0.016			
	Sedentary	Reference							
	Playing surface								
	Synthetic material	1.374 [0.391; 2.360]	0.502	2.74	3.952 [1.479; 10.566]	0.006			
	Do not know	1.886 [0.331; 3.460]	0.804	2.35	6.595 [1.365; 31.858]	0.019			
	Artificial grass	Reference							
Muscular						*0.044*	*0.020*	*4.07*	*0.563*
	Sex								
	Female	0.559 [0.020; 1.100]	0.275	2.03	1.750 [1.020; 3.001]	0.042			
	Male	Reference							
Neck						*0.049*	*0.122*	*3.88*	*0.697*
	BMI	0.294 [0.024; 0.564]	0.138	2.13	1.340 [1.020; 1.758]	0.033			
Arm (post.)						*0.016*	*0.249*	*5.80*	*0.798*
	Padel weekly training	0.945 [0.204; 1.690]	0.378	2.50	2.570 [1.230; 5.401]	0.012			
Elbow						*<0.001*	*0.106*	*19.8*	*0.659*
	Padel Years	-0.140 [-0.270; -0.011]	0.066	-2.12	0.869 [0.763; 0.990]	0.034			
	Presence of an instructor								
	Yes and monitored	2.213 [0.195; 4.232]	1.030	2.15	9.147 [1.215; 68.883]	0.032			
	Yes and non-monitored	2.851 [0.770; 4.932]	1.062	2.69	17.307 [2.160; 138.653]	0.007			
	No	Reference							
Wrist						*0.001*	*0.199*	18.4	*0.819*
	Padel Years	-0.449 [-0.833; -0.065]	0.196	-2.29	0.638 [0.435; 0.937]	0.022			
	Sport plus Padel								
	No	1.60 [0.081; 3.121]	0.775	2.07	4.959 [1.085; 22.670]	0.039			
	Yes	Reference							
	Playing surface								
	Artificial grass	-1.651 [-3.319; 0.017]	0.851	-1.94	0.192 [0.036; 1.017]	0.052			
	Synthetic material	-2.621 [-4.333; -0.909]	0.874	-3.00	0.073 [0.013; 0.403]	0.003			
	Do not know	Reference							
Hand/Fingers						*0.036*	*0.094*	*4.40*	*0.721*
	Padel training duration	0.009 [0.002; 0.015]	0.003	2.55	1.008 [1.002; 1.015]	0.011			
Lower Back						*0.037*	*0.140*	*6.61*	*0.696*
	Field side								
	Left	-3.880 [-6.880; -0.889]	1.530	-2.54	0.021 [0.001; 0.412]	0.011			
	Right	-4.960 [-8.360; -1.564]	1.730	-2.86	0.007 [0.000; 0.209]	0.004			
	Do not know	Reference							

**Injury**	**Factor - Level**	**β (95% CI)**	***SE***	***z***	**Odds Ratio (95% CI)**	***P***	**R^2**	**χ^2**	**AUC**
Knee						*0.007*	*0.081*	*12.20*	*0.609*
	Past sport practice	0.002 [0.001; 0.003]	0.001	2.65	1.002 [1.001; 1.003]	0.008			
	Playing surface								
	Artificial grass	-1.763 [-3.05; -0.475]	0.657	-2.68	0.171 [0.047; 0.622]	0.007			
	Synthetic material	-1.599 [-2.89; -0364]	0.630	-2.54	0.202 [0.059; 0.695]	0.011			
	Do not know	Reference							
Lower Leg (post.)						*0.013*	*0.043*	*6.19*	*0.547*
	Padel Years	0.123 [0.027; 0.219]	0.049	2.52	1.131 [1.028; 1.245]	0.012			

Abbreviations: AUC = area under the receiver-operating-characteristic; BMI = body mass index; β = estimated logistic-regression coefficient; χ^2 = chi-square statistic; p = p-value; R^2 = Nagelkerke; SE = standard error; Z = Wald z-statistic.
